# Modelling of integrated scheduling problem of capacitated equipment systems with a multi-lane road network

**DOI:** 10.1371/journal.pone.0251875

**Published:** 2021-06-02

**Authors:** Di Luan, Mingjing Zhao, Qianru Zhao, Nan Wang

**Affiliations:** 1 School of Traffic and Transportation, Beijing Jiaotong University, Beijing, China; 2 Beijing Jiaotong University Haibin College, Cangzhou, China; 3 School of Computer and Information Technology, Beijing Jiaotong University, Beijing, China; Rutgers University, UNITED STATES

## Abstract

The coordination of different container-handling equipment is an important method for improving the overall efficiency of automated container terminals. In the real terminal, we should consider many real-life issues, such as the equipment capacity, the equipment collision, changing lanes in the multi-lane road, and choosing one of container-handling lanes for each container. This paper proposes the integrated scheduling problem of three container-handling equipment with the capacity constraint and the dual-cycle strategy, for simultaneously solving the equipment scheduling problem, the assignment problem of the container-handling lane and the conflict-free route planning problem of automated guided vehicles (AGVs). With the objective of minimizing the ship’s berth time, we propose a mixed-integer programming model based on the space-time network representation method and two bilevel optimization algorithms based on conflict resolution rules. Finally, numerical experiments are conducted to verify the effectiveness of the proposed model and two bilevel optimization algorithms.

## Introduction

In recent years, developing of the international trade and adjusting of the international transport industry have created high demand for transshipment and warehousing of hub-type container terminals. To improve the advantage over others, terminal operators are continually seeking methods to increase productivity and reduce costs. Terminal operators have applied automated handling equipment and have transformed or built some new automated container terminals (ACTs). Previous studies [1–4] proved the automated equipment performed better than the original manned one in improving operational efficiency and reducing labor costs. Also, an investigation [5] had been carried out to evaluate 20 existing ACTs including Port of Rotterdam, Port of Hamburg and Port of Shanghai. It showed that ACTs had obvious advantages over terminals with manned equipment in operation performance, human safety, environmental sustainability, and financial profitability. Because the automated equipment is controlled automatically, the scheduling system is required to assign tasks and generate work orders. Besides, individual scheduling of one equipment may lead to loss of overall efficiency or even to equipment deadlock [6, 7]. Efficient scheduling based on the coordination of multi-stage equipment system is crucial to improve overall performance of the terminal and needs more research.

In conventional ACTs, the coordination of equipment systems is achieved by double-trolley quayside cranes (DQCs), automated guided vehicles (AGVs) and automated rail mounted gantry cranes (ARMGs) and can be divided into the DQC handling link, the horizontal transportation link and the yard equipment handling link. In the operation of actual ACTs, for each operation link, there are specific influencing factors, including the operation strategy, equipment capacity constraints, and the facility type. These influencing factors directly determine the performance of the related operation link, and even affect the overall efficiency of the terminal. In general, the DQC handling link is often the bottleneck of the whole operation process. At present, there are two DQC operation strategies, the single-cycle traditional strategy and the dual-cycle strategy. In the dual-cycle strategy, one main trolley of a DQC completes one container loading task and one container unloading task within one cycle of a round trip. For the dual-cycle strategy, the operation of one cycle is equivalent to that of two cycles under the traditional strategy. Therefore, the dual-cycle strategy can significantly improve the operation efficiency of the DQC handling link. In addition, equipment capacity constraints and the type of AGV road network are the main factors affecting the overall operational efficiency of actual terminals. In fact, the transfer platform of DQCs or the AGV-mate is equipped with limited storage locations, which restricts the potential for improving the efficiency of the DQC handling link or the horizontal transportation link. The multilane AGV road network is commonly used in the current ACTs and offers greater mobility and flexibility. However, this type of road network is also facing more complex traffic conflicts than the traditional road networks. This paper considers these influencing factors mentioned above including the dual-cycle strategy, the equipment with the capacity limit and AGV conflicts in the multilane AGV road network.

In the existing literature on equipment system integrated scheduling in ACTs, the integrated scheduling of DQCs, AGVs and ARMGs is generally transformed into an integrated problem of equipment scheduling and AGV conflict-free routing planning based on simplified scenarios [8]. These simplified scenarios generally do not take into account the equipment with the capacity limit and selection decisions of containers for handling lanes in the container-handling area of the AGV road network, which are the important factors affecting the scheduling problem of the equipment system. In the AGV routing planning problem, many researches have focused on some types of AGV conflicts and AGV conflict-free routing planning in a single-lane AGV network, ignoring unique conflicts at intersections in a multilane network, which greatly reduces the research value for actual operations [9–11].

In this work, we propose integrated scheduling of DQCs, AGVs and ARMGs in the dual-cycle strategy to solve the problems of equipment scheduling, container-handling lane selection and AGV conflict-free path planning, considering the equipment with the capacity limit and AGV conflicts on the multilane AGV road network, which has not been performed in previous literature and is an extension of the existing terminal equipment integrated scheduling problem. To solve this problem, we also design a mixed-integer programming model and two bilevel optimization algorithms, called the conflict resolution rule combination-based bilevel genetic algorithm (CRRC-BGA) and conflict resolution rule combination-based bilevel hybrid genetic tabu search algorithm (CRRC-BHGTSA).

The structure of this paper is as follows. Section 2 provides a view of the research of container-handling equipment scheduling and AGV path planning. Section 3 systematically explains the proposed integrated scheduling problem and introduces the space-time network representation method. Section 4 presents a mixed integer programming model. Section 5 proposes two bi-level optimization algorithms to solve the integrated scheduling problem. Section 6 introduces the experimental process to verify the validity of the proposed model and algorithms. Section 7 summarizes our work and provides suggestions for future research directions.

## Literature review

Integrated scheduling for the cooperative operation of quayside cranes (QCs), AGVs and ARMGs is the research focus in the scheduling field of equipment systems in ACTs. Meersmans and Wagelmans [7] were the first to propose that separating the scheduling of all kinds of equipment is the main reason for equipment conflicts, lock up and low equipment utilization ratios in the process of operation and that it is necessary to carry out integrated scheduling of the equipment; the authors proposed a branch and bound algorithm and a beam search heuristic algorithm for the integrated scheduling problem. Lau and Zhao [6] extended the above problem from the point of view of improving the overall operation efficiency and reducing the QC operation delay and designed a multi-objective mixed-integer programming model with the objective of minimizing the operating time of three types of equipment and the QC operation delay time. On the basis of the above research, Zeng and Yang [12] proposed an integrated algorithm for the integrated scheduling problem with complex constraints, combining the genetic algorithm, simulation technology and neural network model. Luo and Wu [13] investigated an integrated scheduling model of AGVs and YCs during the loading process, aiming to minimize the ship’s berthing time. The B&B algorithm implemented by CPLEX and a proposed adaptive heuristic algorithm were used to solve small-sized problems and large-sized problems, respectively. Kizilay et al. [14] studied the joint optimization of the scheduling problem of three types of equipment as well as yard location assignments, considering import and export containers, the safety distance and the interference constraints for the quay cranes. Several constraint programming models were proposed to minimize the turnover times of the vessels and maximize terminal throughput. Zhou et al. [15] developed a mixed-integer programming model and a tabu search-based two-stage heuristic algorithm to obtain a comprehensive scheme that included YC schedules and vehicle parking positions. The objective was to minimize the makespan, considering the Chebyshev movement of the yard crane. The above studies focus on the integrated scheduling of three kinds of equipment in a single loading or unloading process and do not take into account the process with the dual-cycle strategy.

The dual-cycle strategy of container-handling equipment in ACTs was first proposed by Goodchild and Daganzo [16] and has been proven to be effective in improving the efficiency of the quayside crane handling link. Then, Chen et al. [17], inspired by this research, defined the integrated scheduling problem of three kinds of equipment in the dual-cycle strategy as a hybrid flow shop scheduling problem (HFSS-B) with priority and congestion constraints and proposed an improved tabu search algorithm. Luo and Wu [18] solved the integrated problem of three kinds of equipment in the dual-cycle strategy, combining equipment scheduling and container storage location allocation in storage yards by a new mixed-integer programming model and an improved genetic algorithm. Zhang et al. [19] proposed an integrated approach based on cycle-time models and the queuing theory, to simulate the coordination of the entire container handling system with double cycling. The approach quantified the long-term effect of the mixed storage strategy on terminal operations. Zhang et al. [20] developed a queuing model with generally distributed service time to model the multiple-process terminal handling system with QC double cycling. The model was used to evaluate the productivity and stability of a terminal system with three kinds of equipment and illustrate the benefit and cost reductions achieved by applying double cycling into the system.

The AGV integrated scheduling problem of scheduling and route planning, in which actual capacitated equipment, space and time constraints are taken into consideration, is considered in a review literature by Vis [21] to be a practical and challenging research direction in the research field of AGV system scheduling that requires further research. Miyamoto [22] proposed that AGV anticollision concerns and the capacity of machine buffers should be considered in the AGV scheduling and routing problem, inspired by the practical application of multi-AGV systems in manufacturing, promoting the extension of the traditional equipment integrated scheduling problem to the integrated problem of capacitated equipment scheduling and AGV conflict-free routing planning. At present, in the research field of handling equipment integrated scheduling in ACTs that combines capacitated equipment system scheduling and AGV conflict-free routing planning, there are a small number of papers focusing on the capacity limit of auxiliary lanes and the conflicts at nodes or intersections on a single-lane road network, which provide important inspiration for our work. Yang et al. [10] designed a mixed-integer programming model and proposed a bilevel genetic algorithm based on congestion prevention rules to obtain a comprehensive scheduling scheme including the equipment scheduling and AGV conflict-free paths, with the goal of minimizing the ship operating time in the port. Ji et al. [11] studied the integrated scheduling problem of double-trolley quayside cranes, AGVs and ASCs in the dual-cycle strategy, considering the node capacity limit, node conflict and overtaking conflict on the road network, and designed a bilevel adaptive genetic algorithm based on a conflict resolution strategy. Zhong et al. [23] solved the integrated scheduling problem of equipment scheduling and AGV dynamic path planning based on conflict and deadlock detection involving quayside cranes, AGVs and rail-mounted gantry (RMG) cranes by a proposed mixed-integer programming model and designed a hybrid genetic-particle swarm optimization algorithm.

The main contributions of this paper are as follows: (1) a mixed-integer programming model based on the space-time network representation method is established to analyze a comprehensive scheduling scheme that integrates the scheduling of three kinds of container-handling equipment, the handling lane selection decision of containers and AGV conflict-free routing planning. This model focuses on the factors that play important roles in practical cooperative operation and that have not been considered in the current research, including the capacity limit for handling equipment and AGV conflicts on multilane AGV road networks. (2) Two bilevel optimization algorithms are proposed, called CRRC-BGA and CRRC-BHGTSA. In both algorithms, the conflict resolution rule combination is designed to solve three types of AGV conflicts on a multilane AGV road network.

## Problem description and space-time network representation method

### Problem description

**Fig 1:**
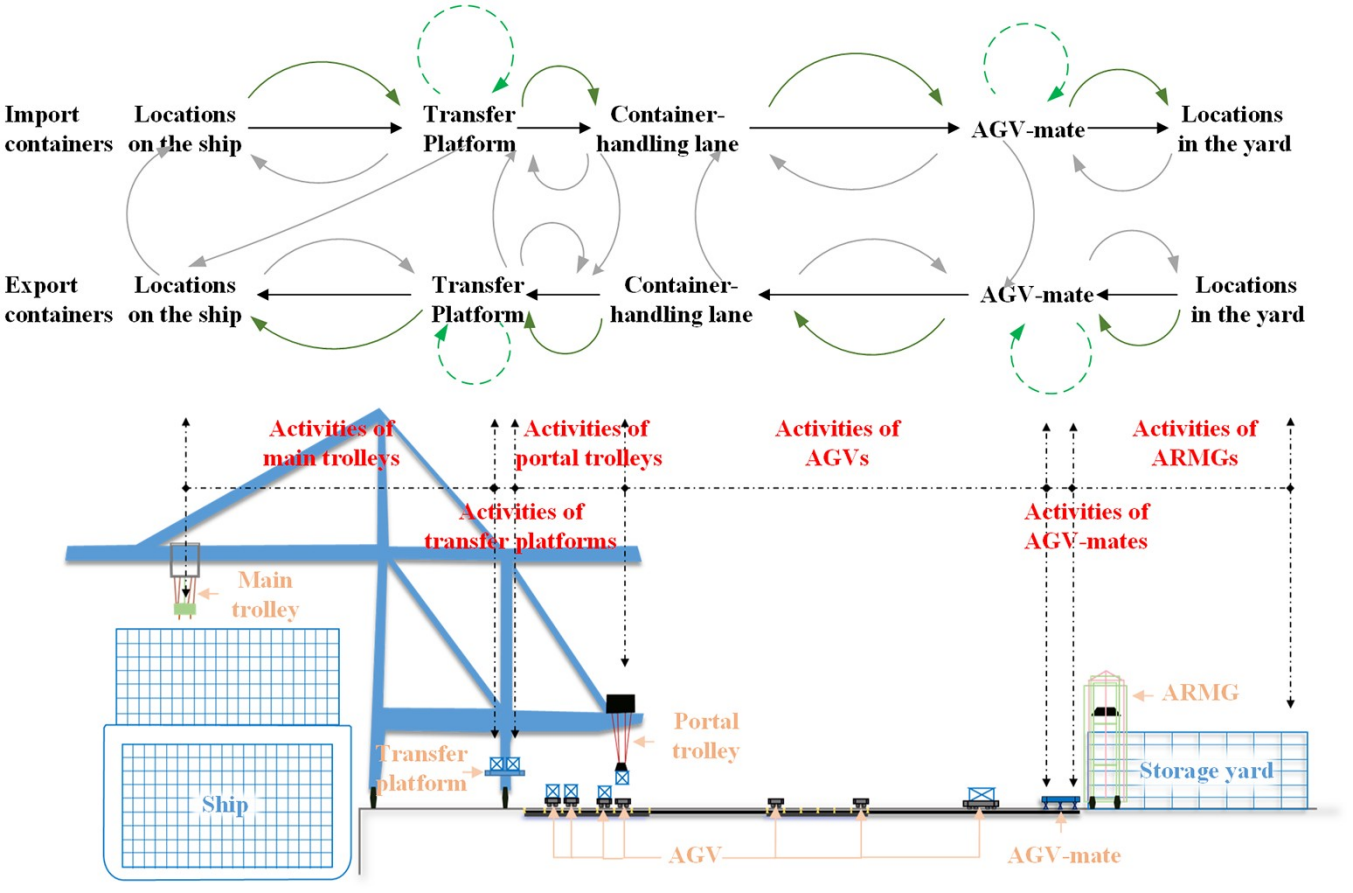
The operation process of equipment systems in ACTs.

The integrated scheduling problem studied in this paper is the obtaining of a comprehensive scheme that includes the scheduling of three types of equipment, the handling lane selection of containers and AGV conflict-free paths. In this problem, the container-handling operation plan of the container ship is given in advance, confirming the information of the containers on board to be unloaded and those from the storage yard to be loaded, including the container serial number, initial locations and destination locations on the ship or in the yard, and the assigned DQCs. During the whole working process, the equipment system is composed of DQCs (including main trolleys, transfer platforms and portal trolleys), AGVs, AGV-mates, and ARMGs and is affected by the service capacity restriction of the container-handling location of some equipment and the function restriction of the multilane AGV road network. The collaboration process of capacitated equipment system is shown in Fig 1.

Each import container is moved by the main trolley in the assigned DQC from its location on the ship to the transfer platform, and then the container is handled by the platform to remove its lockpin. After that, the container is transferred by the portal trolley from the transfer platform to the container-handling lane in the container-handling area of the AGV road network. Each DQC has a fixed area in the container-handling area of the AGV road network, including four container-handling lanes, and one of them needs to be assigned to each container task in the scheduling problem. When the assigned AGV arrives at the corresponding handling lane, the AGV assists the portal trolley in unloading the containers onto the vehicle, moves to the AGV-mate in the corresponding import zone of the yard, and transfers the container to one of the slots on the AGV-mate. Finally, the assigned ARMG transfers the container from the AGV-mate to the yard and unloads it on the given container location. The operation process for each export container is opposite that of each import container.

In this paper, the dual-cycle strategy is adopted in the whole process. The strategy is the rules of the operation sequence of handling import and export containers of DQCs and requires flexible arrangement of the operation sequence, rather than a strict requirement that one cycle of import-container operation and one cycle of export-container operation must be connected back to front [18, 24].

The capacity limitation of the equipment system refers to the fact that the number of container-handling locations on one transfer platform or one AGV-mate is limited. In general, one transfer platform is equipped with two container-handling locations, while one AGV-mate is equipped with five container-handling locations. This means that for a transfer platform or AGV-mate, the number of containers it can hold at any one time and the number of container-handling operations that are allowed in its collaboration with other equipment both have definite quantitative constraints.

**Fig 2:**
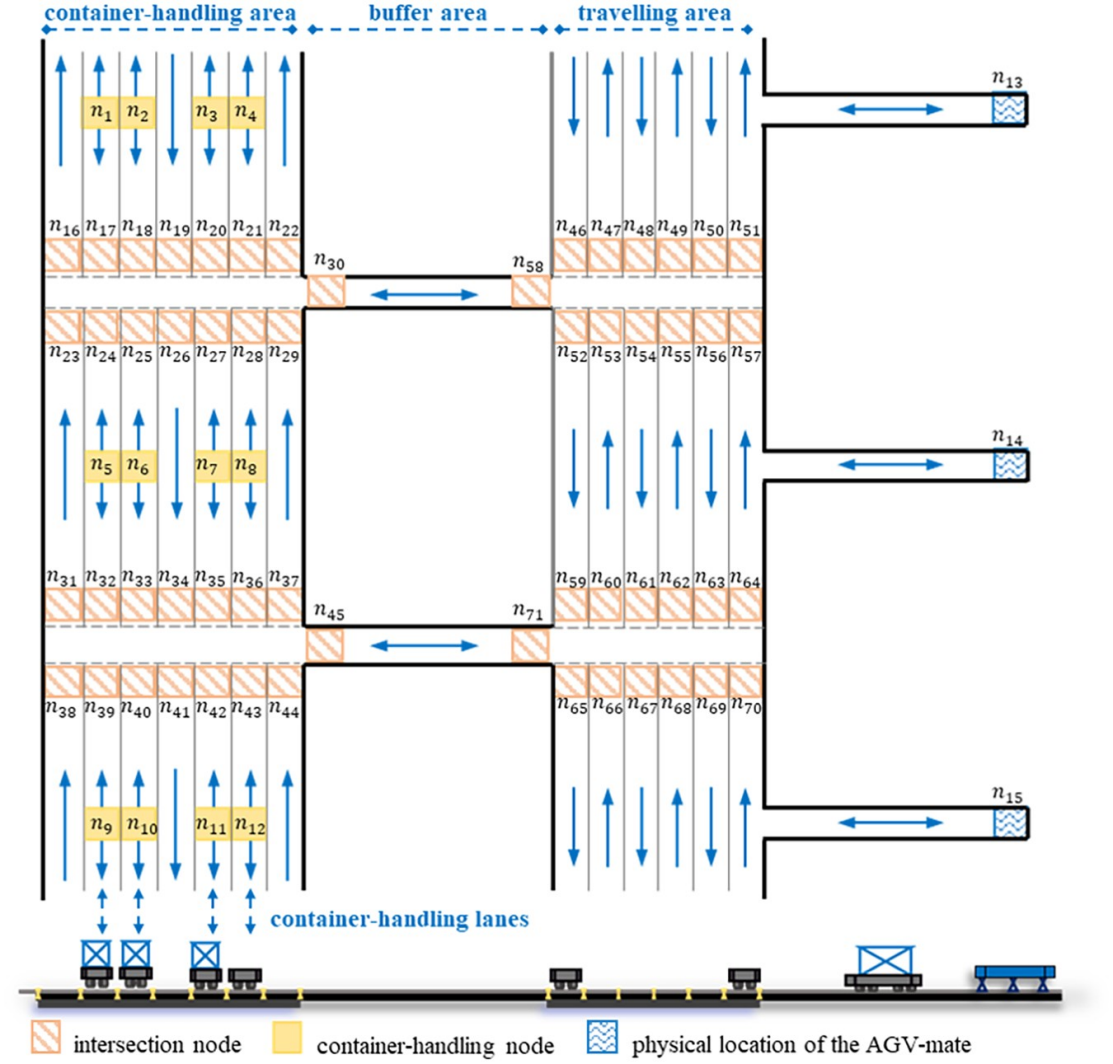
The layout of the multi-lane AGV road network.

The multilane AGV road network studied in this paper is composed of one-way lanes, two-way lanes and intersections and can be divided into three parts, the container-handling area, buffer area and travelling area, as shown in Fig 2. For the vehicle driving process in the road network, many studies focus on the microscopic traffic flow model and the vehicle routing planning problem. Some microscopic traffic flow models [25, 26] have been proposed to study the vehicular movement in the inner area of the intersection, where micro influencing factors are analyzed, such as the driving behavior, the vehicle trajectory and the vehicle size. Zhao [25] designed a two-dimensional vehicular movement model based on optimal control to describe the vehicular movement at intersections without structured and pre-determined lanes. The model can simulate the driving behavior scheme and the distribution of the vehicle trajectory, and can be used to evaluate vehicle conflicts and optimize traffic control strategies at intersections after being extended. However, it is the vehicle routing planning problem that is studied in this paper. In previous studies [27, 28], the vehicle routing planning problem mainly focused on influencing factors related to the nature of the connected network, such as capacitated nodes and the routing path. Micro-influence factors mentioned above were not taken into account. Therefore, in the paper, it is assumed that the AGV is a particle without the consideration of the size, and that the movement of the AGV between any two adjacent nodes follows a fixed guide-path. The road network has the following characteristics:

1. in the container-handling area, container-handling lane groups are set up, each corresponding to a DQCs group. Containers assigned to each DQCs group need to be assigned one lane in the corresponding container-handling lane group by equipment scheduling, and the lane is equipped with the physical location where the related portal trolley and AGV handles this container in a cooperative relationship.
2. the travelling area of the road network connects to the AGV-mate physical location, which sets up five slots for AGVs and the maximum number of AGVs allowed to enter the node corresponding to the location is 5 (the maximum number of AGVs allowed to enter other node is 1) [29].
3. in the road network, there are two types of intersections, namely, the intersection of container-handling area and the intersection of travelling area, both of which connect more lanes and have more travel links through the intersection than urban intersections in the city road network [28].
4. the buffer area consists of a number of parallel two-way single lanes. These two-way lanes are connected to intersection of container-handling area and the intersection of travelling area. AGVs located on the node of the two-way lane can wait at the node before entering the container-handling area or the travelling area to avoid vehicle conflicts at the intersection ahead. In addition, only one AGV is allowed on the node of the lane at the same time.
5. there are three types of AGV conflicts in multi-lane AGV road network, such as the node conflict, the opposite-direction link conflict on two-way roads and the crossed link conflict at intersections [30–32].

This complex multilane AGV road network adds the selection decision of the container-handling lanes to the integrated scheduling problem as one subproblem and poses a greater challenge for the subproblem of AGV conflict-free path planning. In the AGV conflict-free path planning subproblem, the detection methods and resolution strategies of the node conflict and the opposite-direction link conflict on two-way roads have been studied in single-lane road networks or simple multilane road networks and can also be used in complex multilane AGV road networks in this paper [11, 23, 33]. However, crossed link conflict at intersections has not been studied in multilane AGV road networks and is an important problem in multi-AGV conflict-free routing planning. We propose a special conflict resolution rule for this in the algorithms introduced in section 5.

### Space-time network representation method

In this section, we describe in detail the proposed space-time network representation method for modelling the above planning problems. The method includes special structures that can be used without loss of generality to accurately represent the various handling activities, dynamic changes in equipment capacity utilization, cooperative relations among the multiple equipment, and AGV conflicts on the road network.

#### The physical network

**Fig 3:**
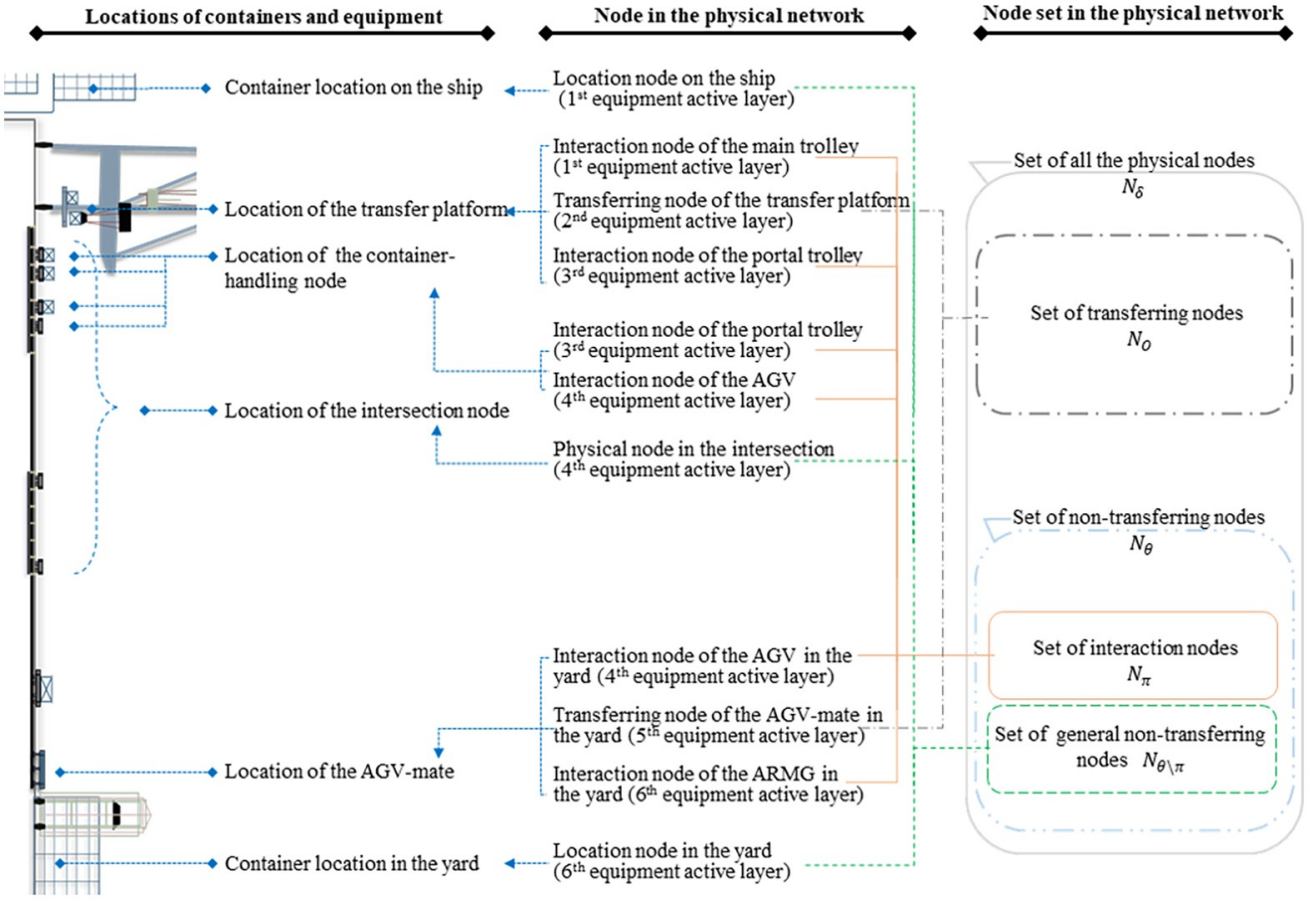
The node and the node set in the physical network.

In the physical network *G* = (*N*_*δ*_, *L*), node *i* ∈ *N*_*δ*_ represents the container location on the ship or in the yard and the physical location where the equipment is operated, and physical link (*i*, *j*) ∈ *L* represents the travelling path of one container or one piece of equipment from node *i* to node *j*. In the physical network shown in Fig 3, activities of each type of equipment occurs at nodes of one certain type or nodes of several certain types and each type of the node corresponds to only one type of equipment. For each type of equipment, all types of nodes where the related activities take place jointly constitute the active region of this type of equipment, and this activity region does not share any common node with that of other types of equipment. Therefore, the physical network is further divided into six equipment active layers according to the active regions of six types of equipment. For the *m*-th equipment active layer, the subnetwork corresponding to this active layer consists of nodes and links that represent available locations and various activities of the *m*-th type of equipment, respectively. In addition, the two subnetworks of two adjacent active layers are linked to each other through a virtual link that represents container transfer.

In this physical network, a unique structure is designed to model the physical location of the transfer platform, the physical location of the AGV-mate and the loading and unloading points of the container-handling lane on the AGV road network. These physical locations, where two or more types of equipment work together for the same container through their own independent activities, are represented by multiple nodes, with each node representing the location and activity of one type of equipment. According to the activity characteristics of the corresponding equipment, those nodes are classified into the following two categories:

1. the transferring node, represents the physical location of the fixed equipment (such as the transfer platform and the AGV-mate), and the set of all of them is defined as *N*_*o*_. Transferring nodes are further subdivided into two types, namely the transferring node of the transfer platform and the transferring node of the AGV-mate in the yard;
2. the interaction node, represents the positions of other mobile equipment staying in the above-mentioned physical locations, and the set of all of them is defined as *N*_*π*_. Interaction nodes are further subdivided into some types, namely the interaction node of the main trolley/ portal trolley, the interaction node of the portal trolley/ AGV in the container-handling area and interaction node of the AGV/ ARMG in the yard.

**Fig 4:**
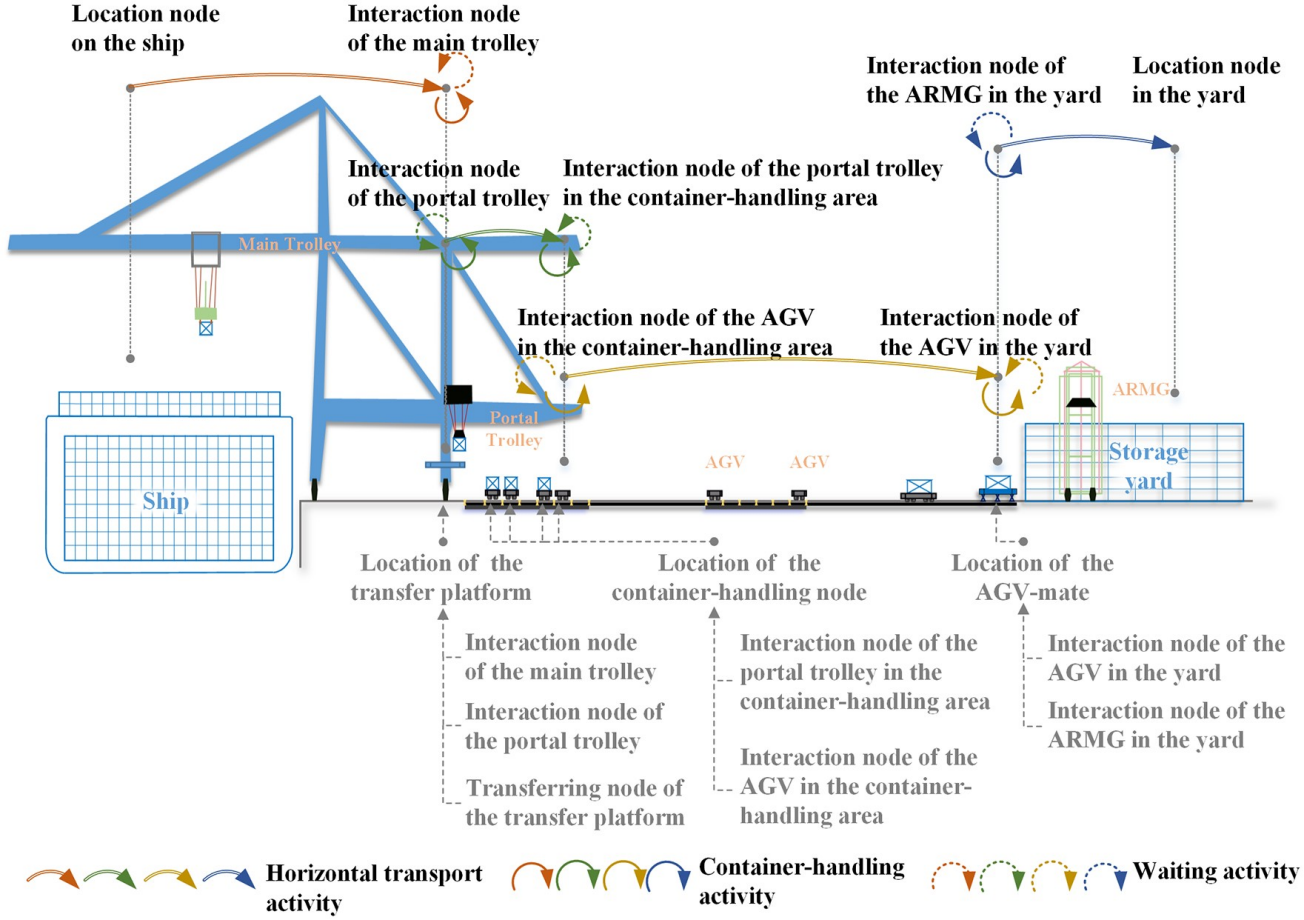
Equipment activities that occur on six types of interaction nodes.

Although the six types of interaction nodes are associated with different types of equipment, these equipment activities that occur on these interaction nodes are of the same type. As shown in Fig 4, these equipment activities include the horizontal transport activity, the container-handling activity and the waiting activity. For any type of the interaction node, one related equipment arrives the node through a horizontal transport activity, then may wait for a period of time at the node, or may directly cooperate with other equipment for one container-handling activity, and finally leaves the node through another horizontal transport activity. In addition, these nodes share same operation logic constraints and capacity constraints. Therefore, these nodes are considered as a class of nodes with same properties and put into one node set to make it clearer and more concise in the proposed physical network and the mathematical model. So do these transferring nodes and other nodes. In this physical network as shown in Fig 3, these nodes except transferring nodes, all represent the physical location that mobile equipment can reach and share some same constraints. Therefore, they are put into the same node set defined as the non-transferring node set, represented by *N*_*θ*_. In addition, the non-transferring node set consists of interaction nodes and other nodes. These two types of nodes are very different in equipment activities and operation logic constraints and should be divided into two subsets to make it more distinct. In the non-transferring node set *N*_*θ*_, for nodes except interaction nodes, define the node set as the general non-transferring node set, represented by *N*_*θ*\*π*_. Then we have *N*_*θ*_ = *N*_*π*_ ∪ *N*_*θ*\*π*_.

#### Construction of the space-time network

Based on these nodes and links in the physical network mapped to these activities of six types of equipment, the planning horizon *T*_*max*_ (such as 3 hours) is discretized into time periods *T* = {1, …, *T*_*max*_}, and a space-time network graph $G^{T}=(N_{\delta }^{T},A_{\delta })$ consisting of 6 equipment active layers is constructed. In the space-time network, the space-time node (*i*, *t*) represents the physical node *i* ∈ *N*_*δ*_ at time *t*. The physical node *i* represents the location on the ship or in the yard and the physical location where the equipment can reach in the physical network. And *i*_0_, *i*_1_, *i*_2_,…, represent nodes marked with different ordinal numbers in the physical network. In addition, the space-time active arc *a*_(*i*,*j*,*t*,*t*′)_ ∈ *A*_*σ*_ not only represents the actual space position change during the real activity of the equipment, but also represents a kind of relationship mapping to the connection between different positions at the same time or between the same position at different times. So, sometimes, some of these arcs are virtual. In general, there are two types of space-time active arcs, i.e., space-time active arcs within one equipment active layer and those between two adjacent equipment active layers.

The space-time active arc within one equipment active layer *a*_(*i*,*j*,*t*,*t*′)$,\;i,j\in N_{\delta }^{m}$_, represents the activity of the corresponding equipment within the scope of their active nodes. In this space-time network, we define space-time activity arcs in the *m*-th equipment active layer as non-transferring active arcs with the set $A_{\theta }^{m}$ and then represent the set of those in all active layers by *A*_*θ*_. The non-transferring active arcs *a*_(*i*,*j*,*t*,*t*′)_ can be subdivided into the following four types according to the types of corresponding activities:

1. the horizontal transport active arc, $i,j\in N_{\delta }^{m}$, *i* ≠ *j*, represents one transportation activity from physical node *i* to *j* in the time range *t*~*t*′. The length of the space-time arc |*a*_(*i*,*j*,*t*,*t*′)_| depends on the travel time *TT*(*i*, *j*) between the two physical nodes.
2. the container-handling active arc, $i=j,i\in N_{\delta }^{m}$, represents one container loading or unloading activity at node *i* within a time range *t*~*t*′. Container handling active arcs corresponding to different entity positions have different fixed lengths, which are determined by node *i*.
3. the waiting active arc, $i=j,i\in N_{\delta }^{m},t^{\prime }=t+1$, represents one waiting activity at physical node *i* within 1 unit time range *t*~(*t* + 1). The length of the waiting active arc |*a*_(*i*,*i*,*t*,*t*+1)_| is 1. Therefore, each waiting activity lasting *n* (*n* is a positive integer and greater than 1) units of time in the real world can be represented by *n* adjacent waiting active arcs.
4. the container-lockpin handling active arc, $i=j,i\in N_{\delta }^{2}$, indicates one container-lockpin removal or installation activity for one container on node *i* of the transfer platform within time range *t*~*t*′. The time required for the removal and installation of the lockpin is fixed, so the length of the container-lockpin handling active arc |*a*_(*i*,*j*,*t*,*t*′)_| is fixed.

The space-time active arc between two adjacent active layers, $a_{\left(i,j,t,t^{\prime }\right)},i\in N_{\delta }^{m},j\in N_{\delta }^{m^{\prime }}(m^{\prime }\in \{m+1,m-1\}),\;t=t^{\prime },$ represents the result of one container transferring activity from one piece of equipment to another at the completion time. We define the space-time active arc between any two adjacent active layers as the transferring active arc with the arc set *A*_*O*_. And the transferring active arc appears together with two related container-handling active arcs. In the space-time network, one transferring active arc and two container-handling active arcs work together to represent one actual loading or unloading activity. Since the fixed length of two container-handling active arcs is designed to indicate the duration of the loading or unloading activity, the transferring active arc is designed to only represent the result of the container transfer. Therefore, the transferring active arc is defined to be virtual and it does not have a duration.

In the space-time network, the method of determining the sequence number of six equipment active layers obeys the rules of the operation sequence of handling an import container by the equipment system, and the 1^st^~6^th^ layers correspond to the main trolleys, transfer platforms, portal trolleys, AGVs, AGV-mates and ARMGs. For any container *k*, the operation performed on it by the *m*-th type of equipment corresponds to one transfer process from physical node *i* to physical node *j* within the equipment active range. For container *k*, we define these two nodes, node *i* and node *j*, as its task starting point *O*^*m*^(*k*) and its task end point *D*^*m*^(*k*) at the *m*-th equipment active layer, respectively. Therefore, the whole operation process of container *k* in the space-time network is defined as the path that starts at the space-time node corresponding to the initial container location and ends at the space-time node corresponding to the destination container location, in turn going through the space-time node corresponding to each task start point and each task end point on six equipment active layers. The path includes space-time active arcs within any equipment active layer, such as horizontal transport active arcs, container-handling active arcs, waiting active arcs and container-lockpin handling active arcs, and transferring active arcs between any two adjacent active layers.

**Fig 5:**
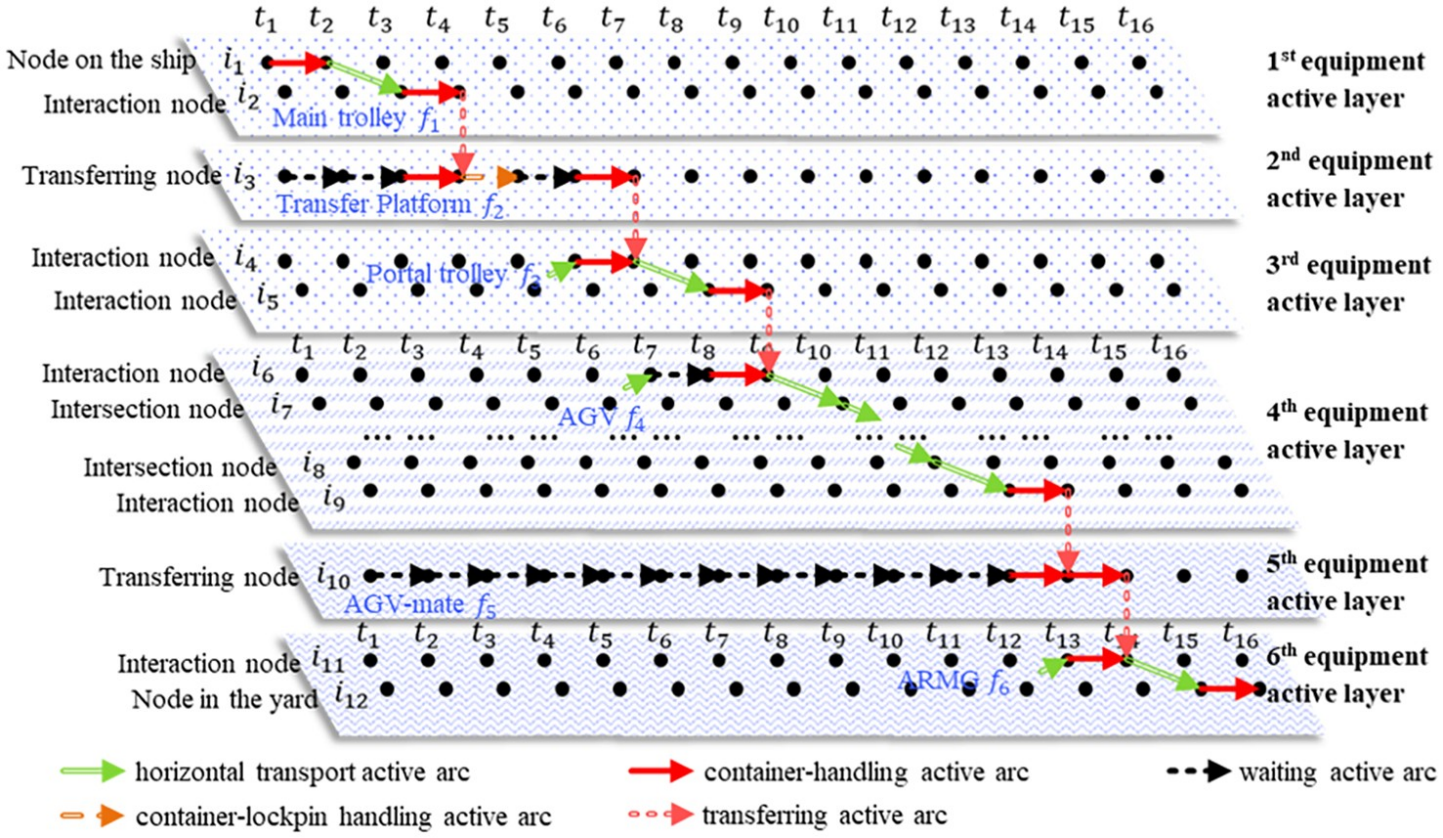
An example of the space-time representation of the container-importing process.

Fig 5 illustrates a simple space-time network with one container location on the ship, one container location in the yard and 16 time periods, where one piece of equipment of each type is used. The example shows the path of import container *k*_0_ from the container location node *i*_1_ on the ship to the container location node *i*_12_ in the yard. For container *k*_0_, the main trolley of the assigned DQC loads the container at node *i*_1_ (task start point *O*^1^(*k*)), then transports the container to the interaction node *i*_2_ (task end point *D*^1^(*k*)) at time *t*_3_. Meanwhile, transfer platform *f*_2_ ∈ *FAC*^2^ is assigned to the next step of the container task at time *t*_1_ and wait until the main trolley is also ready for the loading and unloading activity of container *k*_0_. And the main trolley begins unloading it to the transferring node of transfer platform *i*_3_, which is the task start point *O*^2^(*k*) and task end point *D*^2^(*k*) of the container in the second equipment active layer. The transfer platform removes the container lockpin after receiving the container in the time range *t*_4_~*t*_5_ and waits for the assignment portal trolley *f*_3_ ∈ *FAC*^3^ in the time range *t*_5_~*t*_6_. Subsequently, the portal trolley *f*_3_, AGV *f*_4_ ∈ *FAC*^4^, AGV-mate *f*_5_ ∈ *FAC*^5^ and ARMG *f*_6_ ∈ *FAC*^6^, in a similar way, work cooperatively and in turn complete the transfer operations in 3^rd^~6^th^ equipment active layers of container *k*_0_, carrying the container through the path, *i*_3_ → *i*_4_→ *i*_5_→ *i*_6_→ *i*_7_→ *i*_8_→ *i*_9_→ *i*_10_→ *i*_11_→ *i*_12_ and unloading it at the node *i*_12_, which corresponds to the final container location in the yard.

#### Time-space representation of container-handling cooperative operation

Equipment cooperative loading and unloading activities mainly take place at the transfer platform physical location or AGV-mate physical location and are accomplished with two or more types of equipment. In the space-time network representation method, a special structure is designed using two container-handling active arcs and one related transferring active arc to represent the operation of each piece of equipment and the change in the carrier carrying the container at the end time of the loading and unloading operation. In the representation method, the capacity constraints of the transfer platforms and AGV-mates are considered, and the number of space-time active arcs linked by one space-time node is designed to indicate the capacity utilization of one actual location or one piece of equipment corresponding to the space-time node. In this scenario, the number of container locations on the transfer platform is 2 and that on the AGV-mate is 5. We take the transfer platform as an example for illustration. For transfer platform *f*_0_, the transferring node of transfer platform *i*_0_ corresponds to the space-time nodes (*i*_0_, *t*), *t* ∈ *T*. For each of the space-time nodes, the maximum quantity of transferring active arcs that it can link represents the maximum capacity for handling the containers of the transfer platform, and for each of the space-time nodes and the time of the node, the maximum quantity of arcs operating at the time (including container-handling active arcs, container-lockpin handling active arcs and transferring active arcs) represents the maximum capacity of containers on the transfer platform; in this example, both of the maximum quantities are 2.

**Fig 6:**
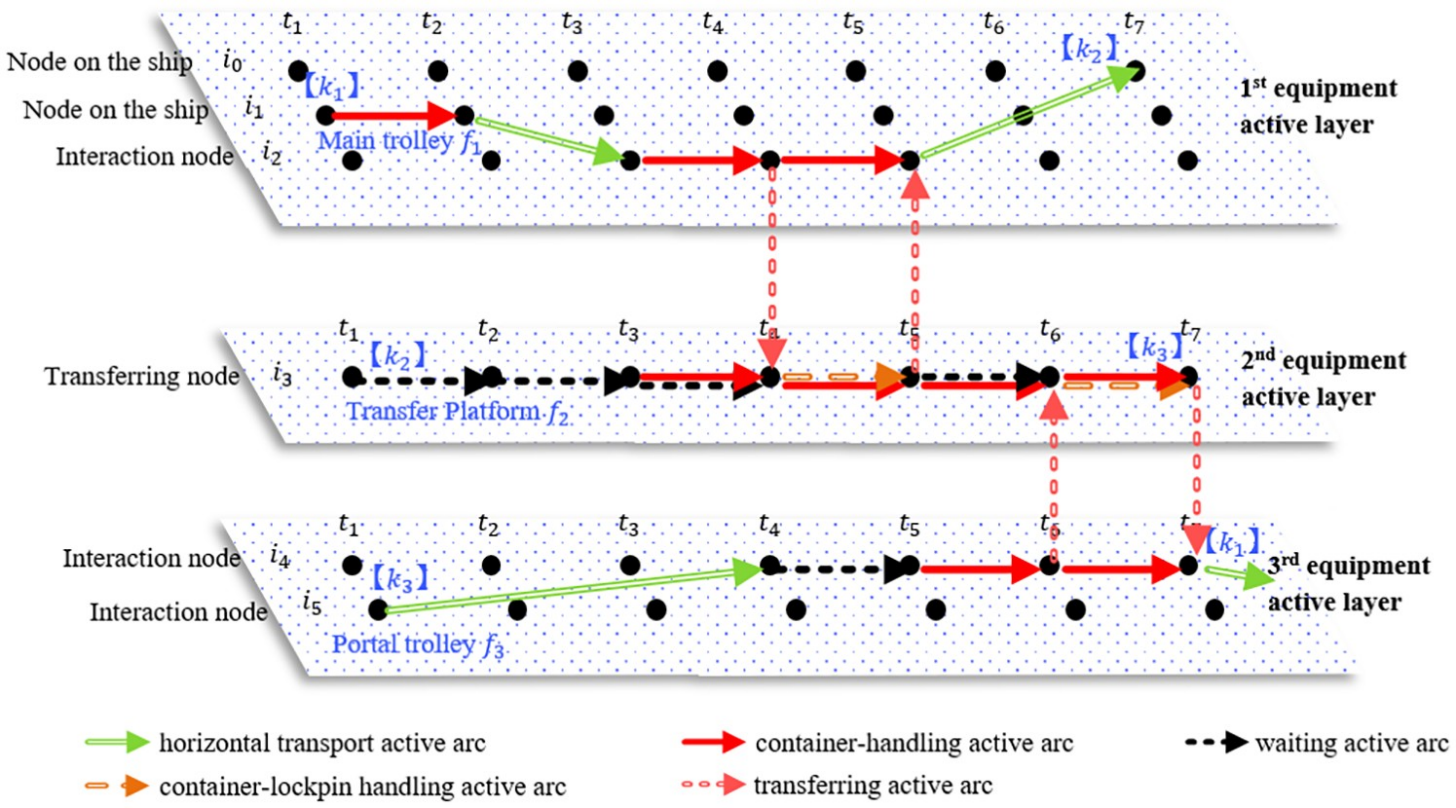
An example of the space-time representation of the cooperative operation of the DQC.

Fig 6 shows a cooperative operation for handling three containers with three types of equipment at the physical location of a double-trolley quayside crane. This example shows the paths of three containers, import container *k*_1_ and export containers *k*_2_ and *k*_3_. The import container *k*_1_ is transferred from node *i*_1_ to node *i*_2_ ∈ *N*^*M*^ by the designated main trolley *f*_1_ ∈ *FAC*^1^, in turn going through a container-handling active arc $a_{\left(i_{1},i_{1},t_{1},t_{2}\right)}$ and a horizontal transport active arc $a_{\left(i_{1},i_{2},t_{2},t_{3}\right)}$. At time *t*_3_, for space-time node (*i*_3_, *t*_3_), which corresponds to the assigned transfer platform *f*_2_ ∈ *FAC*^2^, the number of transferring active arcs linked to the space-time node is 0, and the number of active arcs (including container-handling active arcs, container-lockpin handling active arcs and transferring active arcs) operating at the time and corresponding to the space-time node is 1. This means that transfer platform *f*_2_ has a free location for one container. Then, the main trolley *f*_1_ and transfer platform *f*_2_ simultaneously carry out the loading and unloading operation to transfer container *k*_1_ from the main trolley to the transfer platform in the time range *t*_3_~*t*_4_. The process is represented by two container-handling active arcs $a_{\left(i_{2},i_{2},t_{3},t_{4}\right)},a_{\left(i_{3},i_{3},t_{3},t_{4}\right)}$ and one transferring active arc $a_{\left(i_{2},i_{3},t_{4},t_{4}\right)}$. Container *k*_1_ enters space-time node (*i*_3_, *t*_4_) at time *t*_4_, then goes through container-lockpin handling active arc $a_{\left(i_{3},i_{3},t_{4},t_{5}\right)}$ and waiting active arc $a_{\left(i_{3},i_{3},t_{5},t_{6}\right)}$ and again receives one loading and unloading operation in the time range *t*_6_~*t*_7_ entering space-time node (*i*_4_, *t*_7_). In this process, the number of containers on transfer platform *f*_2_ at time *t*_4_ reaches the upper limit ${Cap}_{f}^{2}$ because of the operation of container *k*_1_ and container *k*_2_ on transfer platform *f*_2_, and transfer platform *f*_2_ can no longer serve more containers. Therefore, after entering space-time node (*i*_4_, *t*_4_), container *k*_3_ needs to wait until time *t*_5_ when transfer platform *f*_2_ is available. Similar to container *k*_1_, export containers *k*_2_ and *k*_3_ reach space-time nodes (*i*_0_, *t*_7_) and (*i*_3_, *t*_7_), respectively, through various space-time activity arcs in the time range *t*_1_~*t*_7_.

**Fig 7:**
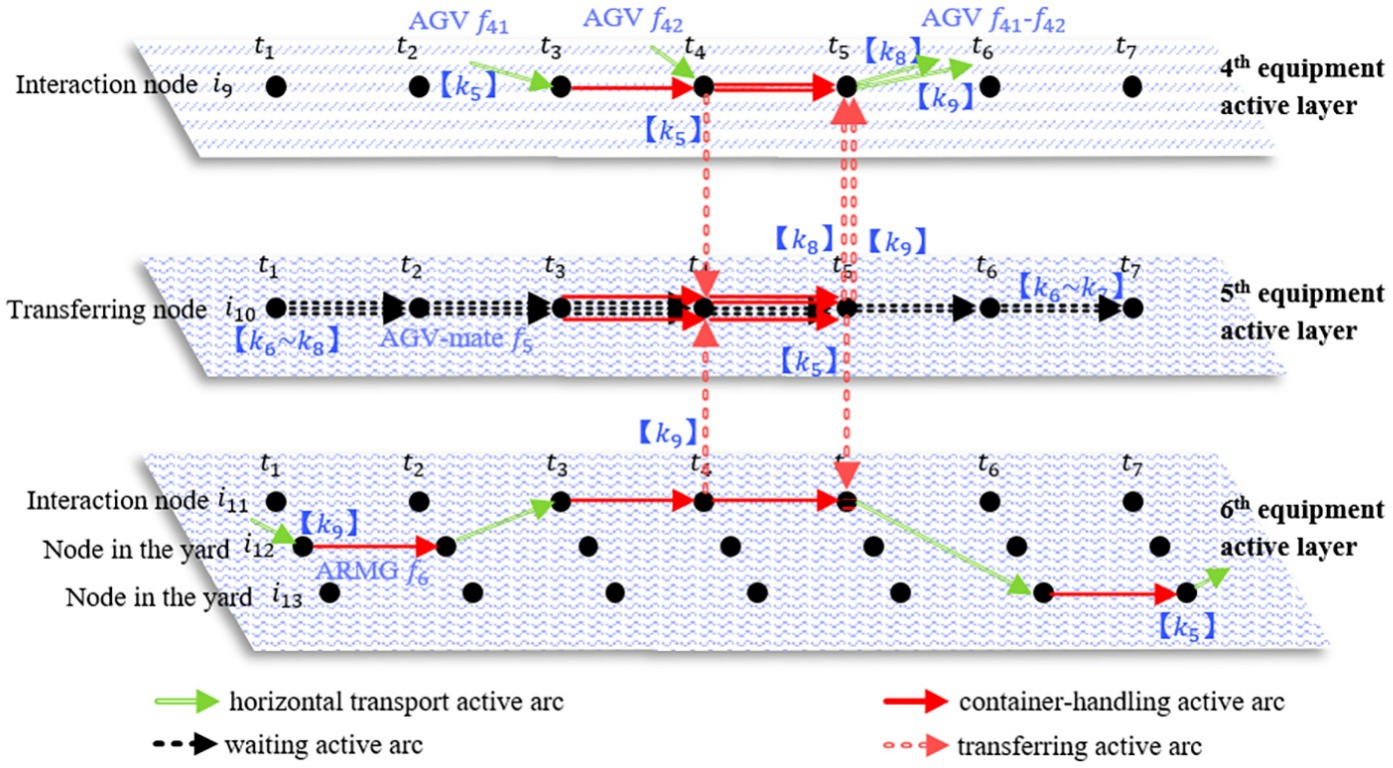
An example of the space-time representation of the cooperative operation corresponding to the AGV-mate.

In the space-time network, the same method is used to model the equipment cooperative loading and unloading activities at the physical location of the AGV-mate. Fig 7 shows the collaborative operation of five containers carried out by two AGVs, an AGV-mate and an ARMG. This example shows the paths of five containers in the space-time model, including those of import container *k*_5_ and export containers *k*_6_~*k*_9_. This example is similar to that in the Fig 6 and does not require further elaboration. In particular, because the loading and unloading activities at the transfer platform are different from those at the AGV-mate, there are two main differences in time-space representation of the two related collaborative process. Firstly, since the transfer platform is equipped with two container-handling locations and the AGV-mate is equipped with five locations, the transferring node related to the AGV-mate can link more space-time activity arcs in the same time period. In time range *t*_3_~*t*_4_, there are five space-time activity arcs linking the transferring node, including two container-handling active arcs and three waiting active arcs. This means that the AGV-mate is completely occupied by these activities of five containers. Secondly, because the transfer platform needs to remove and install the lockpin for the container and the AGV-mate does not, the transferring node related to the AGV-mate does not link the container-lockpin handling active arc. Take container *k*_3_ for example. After finishing a loading and unloading operation transferring to space-time node (*i*_10_, *t*_4_), container *k*_3_ goes straight to another loading and unloading operation organized by the AGV-mate and the ARMG at time *t*_4_.

#### Time-space representation of AGV conflicts

In the multilane AGV road network, the conflicts that may be caused by multiple AGVs can be subdivided into three types, namely, the node conflict at the same space-time node, the opposite-direction link conflict on two-way roads and the crossed link conflict at intersections. Considering the common characteristics of nodes occupied by various AGV conflicts, a representation method is designed to represent multi-AGV travelling activities on the road network by using the horizontal transport active arc or the waiting active arc *a* and the node set $N_{a}^{T}(a)$ of the space-time nodes occupied by the arc *a*. We can judge whether there are any AGV conflicts between any two AGVs $f_{0},f_{0}^{\prime }\in {FAC}^{4}$ by using the intersection $N_{\cap }^{T}(a_{1},a_{2})$ of the two node sets of the space-time nodes occupied by the two space-time active arcs *a*_1_, *a*_2_ of those AGVs. If $N_{\cap }^{T}(a_{1},a_{2})=\emptyset$, then the two space-time active arcs are relatively independent, and there is no AGV conflict between the two AGVs. Otherwise, the activities of the two AGVs could conflict. The designed method can be used to judge and describe AGV conflicts occurring at road nodes, two-way sections and intersections.

**Fig 8:**
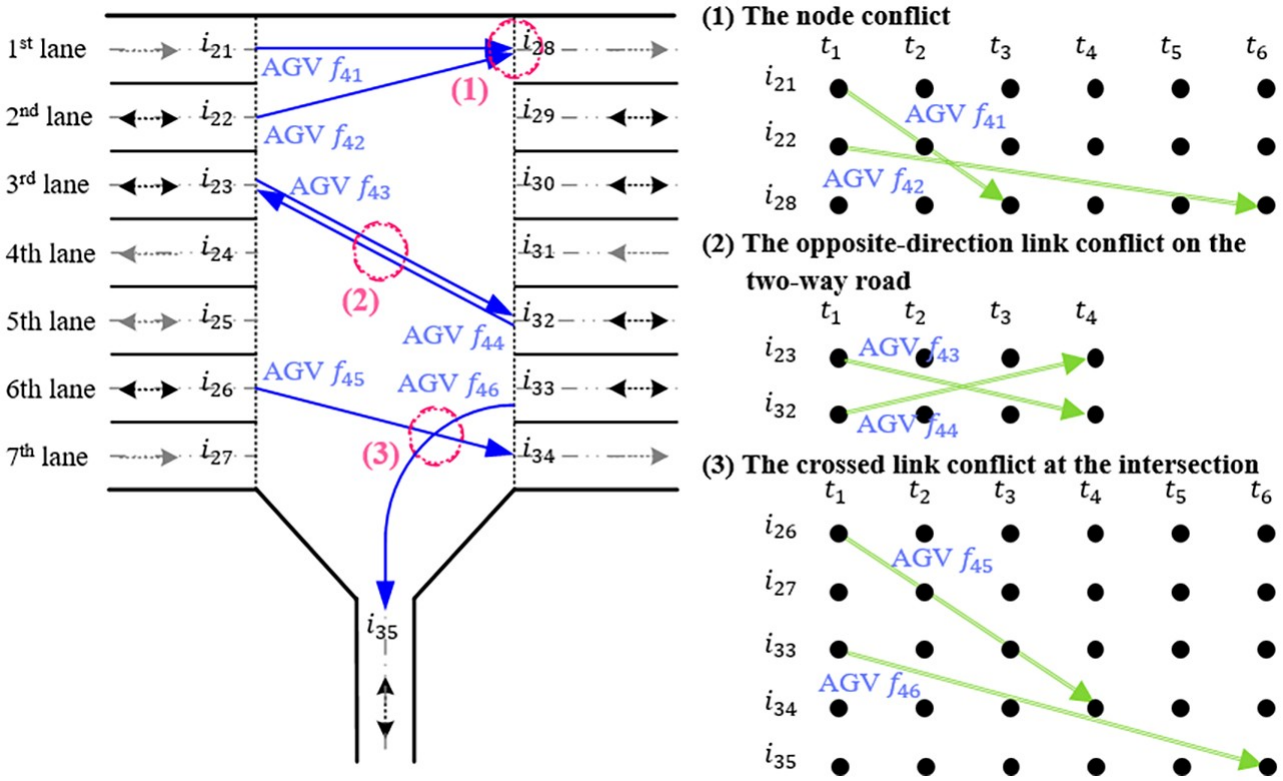
An example of the space-time representation of AGV conflicts at one intersection.

Because intersections involve all types of AGV conflicts, one intersection of the container-handling area is taken as an example to illustrate the above representation method for AGV conflicts, as shown in Fig 8. The physical location of the intersection is represented by space-time nodes corresponding to 15 physical nodes, while the AGV travelling activities crossing the intersection by horizontal transport active arcs link the space-time nodes. This example illustrates three types of AGV conflicts when six AGVs simultaneously travel through the intersection in the time range *t*_1_~*t*_6_. AGV *f*_46_ goes through the horizontal transport active arc $a_{\left(i_{33},i_{35},t_{1},t_{6}\right)}$ occupying the 6th and 7th lanes (represented by links (*i*_26_, *i*_33_) and (*i*_27_, *i*_34_)) and the physical node *i*_35_ corresponding to the end of the active arc, and correspondingly, AGV *f*_45_ goes through the horizontal transport active arc $a_{\left(i_{26},i_{34},t_{1},t_{4}\right)}$ occupying the same lanes. Therefore, the space-time node occupations by the two active arcs of the AGVs are

$$N_{a}^{T}(a_{\left(i_{33},i_{35},t_{1},t_{6}\right)})=\{i_{26},i_{27},i_{33},i_{34},i_{35}\}\times \{t_{1},t_{2},t_{3},t_{4},t_{5},t_{6}\},$$

$$N_{a}^{T}(a_{\left(i_{26},i_{34},t_{1},t_{4}\right)})=\{i_{26},i_{27},i_{33},i_{34}\}\times \{t_{1},t_{2},t_{3},t_{4}\}.$$

Because $N_{\cap }^{T}(a_{\left(i_{33},i_{35},t_{1},t_{6}\right)},a_{\left(i_{26},i_{34},t_{1},t_{4}\right)})\neq \emptyset$, the two horizontal transport active arcs share the same space-time nodes, which means that the travel of the two AGVs *f*_45_ and *f*_46_ causes conflicts. Similarly, this method can be used to judge and describe the node conflict of the same space-time node caused by the travel of AGVs *f*_41_ and *f*_42_, as well as the opposite-direction link conflict on a two-way road caused by that of AGVs *f*_43_ and *f*_44_.

#### Container task

All containers in the scene can be classified as import container *k* ∈ *I* and export container *k*′ ∈ *E*. Each container has been given in advance one given container location node *i*_1_ ∈ *N*^*SS*^ on the ship and one given container location node *i*_2_ ∈ *N*^*YS*^ in the yard as the starting point *O*(*k*_0_) and the end Point *D*(*k*_0_) of the entire process, and each container is assigned to a particular DQC. For All containers, the duration of loading and unloading activities by different equipment in the model depends on the physical location of the handling activities.

## Model formulation

### Model assumptions

1. The container in this paper means 40-foot standard container. According to the capacity limit of the actual equipment, each of these container locations on transfer platform or AGV-mate can serve one standard container, every mobile equipment (including the main trolley, the portal trolley, the AGV and the ARMG) can only transport or load or unload one standard container at a time.
2. The operation time corresponding to the travelling process and the loading or unloading process of the mobile equipment, is the determination value.
3. The transfer platform of the DQC can serve 2 containers at most at the same time to remove or install the container lockpin.
4. AGV travels from one node to another in the road network at the constant speed and may stop and wait at partial nodes in the road network.
5. The container storage area in the yard can be classified into two types, namely the import container storage area and the export container storage area, and each type of container storage area can only load or unload the corresponding type of containers.
6. In every container storage area in the yard, loading and unloading operations for all those containers are performed by one specific ARMG, and each ARMG can only serve one container storage area.

### Model parameters

(1) Parameter sets

$$\begin{array}{ll} N_{\sigma } & \text{Set}\;\text{of}\;\text{physical}\;\text{nodes},\;\text{including}\;\text{transferring}\;\text{nodes}\;i\in N_{O}\;\text{and}\;\text{non-transferring}\;\text{nodes}\;i^{`}\in N_{\theta },N_{\sigma }=N_{O}\cup N_{\theta }\\ N_{\sigma }(f) & \text{Set}\;\text{of}\;\text{physical}\;\text{nodes}\;\text{in}\;\text{the}\;\text{active}\;\text{region}\;\text{of}\;\text{equipment}\;f\\ N_{\pi } & \text{Set}\;\text{of}\;\text{interaction}\;\text{nodes}\;\text{in}\;\text{non-transferring}\;\text{nodes}\\ N_{\sigma }^{m} & \text{Set}\;\text{of}\;\text{physical}\;\text{nodes}\;\text{in}\;\text{the}\;m\text{-th}\;\text{equipment}\;\text{active}\;\text{layer}\\ N_{\pi }^{m} & \text{Set}\;\text{of}\;\text{interaction}\;\text{nodes}\;\text{in}\;\text{the}\;m\text{-th}\;\text{equipment}\;\text{active}\;\text{layer}\\ \Omega_{group}^{w} & \text{Set}\;\text{of}\;\text{physical}\;\text{nodes}\;\text{in}\;\text{the}\;w\text{-th}\;\text{node}\;\text{group}\;\text{in}\;\text{the}\;\text{AGV}\;\text{road}\;\text{network},\;w\in \{1,\ldots ,W_{group}\}\\ \Omega_{cluster}^{w} & \text{Set}\;\text{of}\;\text{physical}\;\text{nodes}\;\text{in}\;\text{the}\;w\text{-th}\;\text{intersection}\;\text{node}\;\text{cluster}\;\text{in}\;\text{the}\;\text{AGV}\;\text{road}\;\text{network},\;w\in \{1,\ldots ,W_{c.luster}\}\\ \Psi_{cluster}^{w,syn}(i,j) & \text{Set}\;\text{of}\;\text{links}\;\text{that}\;\text{can}\;\text{be}\;\text{synchronized}\;\text{with}\;\text{link}\;(i,\;j)\;\text{and}\;\text{also}\;\text{have}\;\text{no}\;\text{conflicting}\;\text{relationship}\;\text{with}\;\text{it}\;\text{in}\;\text{the}\;\text{link}\;\text{set}\\ & \Psi_{cluster}^{w}\;\text{of}\;\text{the}\;w\text{-th}\;\text{intersection}\;\text{in}\;\text{the}\;\text{AGV}\;\text{road}\;\text{network},\;w\in \{1,\ldots ,W_{c.luster}\}\\ \Psi_{cluster}^{w,exc}(i,j) & \text{Set}\;\text{of}\;\text{links}\;\text{that}\;\text{are}\;\text{conflicting}\;\text{and}\;\text{mutually}\;\text{exclusive}\;\text{with}\;\text{link}\;(i,j)\;\text{in}\;\text{the}\;\text{link}\;\text{set}\\ & \Psi_{cluster}^{w}\;\text{of}\;\text{the}\;w\text{-th}\;\text{intersection}\;\text{in}\;\text{the}\;\text{AGV}\;\text{road}\;\text{network},\;w\in \{1,\ldots ,W_{c.luster}\}\\ T & \text{Set}\;\text{of}\;\text{time}\;\text{periods}\;\text{in}\;\text{the}\;\text{planning}\;\text{horizon},\;T=\{1,\ldots ,t_{max}\}\\ FAC^{m} & \text{Set}\;\text{of}\;\text{equipment}\;\text{in}\;\text{the}\;m\text{-th}\;\text{equipment}\;\text{active}\;\text{layer} \end{array}$$

$$\begin{array}{ll} FAC(i) & \text{Set}\;\text{of}\;\text{equipment}\;\text{in}\;\text{the}\;\text{equipment}\;\text{active}\;\text{layer}\;\text{in}\;\text{which}\;\text{physical}\;\text{node}\;i\;\;\text{resides}\\ A_{\sigma } & \text{Set}\;\text{of}\;\text{space-time}\;\text{active}\;\text{arcs}\;\text{in}\;\text{the}\;\text{space-time}\;\text{network},\;\text{including}\;\text{transferring}\;\text{active}\;\text{arcs}\\ & \;a\in A_{O}\;\text{and}\;\text{non-transferring}\;\text{active}\;\text{arcs}\;a^{`}\in A_{\theta },\;A_{\sigma }=A_{O}\cup A_{\theta }\\ A_{\theta }^{i,t} & \text{Set}\;\text{of}\;\text{non-transferring}\;\text{active}\;\text{arcs}\;\text{that}\;\text{occur}\;\text{at}\;\text{physical}\;\text{node}\;i\;\text{but}\;\text{not}\;\text{end}\;\text{at}\;\text{time}\;t\\ In(i,t) & \text{Set}\;\text{of}\;\text{non-transferring}\;\text{active}\;\text{arcs}\;\text{that}\;\text{end}\;\text{at}\;\text{space-time}\;\text{node}\;(i,t)\\ Out(i,t) & \text{Set}\;\text{of}\;\text{non-transferring}\;\text{active}\;\text{arcs}\;\text{that}\;\text{start}\;\text{at}\;\text{space-time}\;\text{node}\;(i,t)\\ Z & \text{Set}\;\text{of}\;\text{import}\;\text{container}\;\text{tasks}\;k\in I\;\text{and}\;\text{export}\;\text{container}\;\text{tasks}\;k^{`}\in E,\;Z=I\cup E\\ S & \text{The}\;\text{dummy}\;\text{starting}\;\text{task}\;\text{in}\;\text{container}\;\text{tasks}\\ F & \text{The}\;\text{dummy}\;\text{ending}\;\text{task}\;\text{in}\;\text{container}\;\text{tasks}\\ O & \text{Set}\;\text{of}\;\text{real}\;\text{container}\;\text{tasks,}\;\text{the}\;\text{dummy}\;\text{starting}\;\text{task}\;\text{and}\;\text{the}\;\text{dummy}\;\text{ending}\;\text{task},\;O=I\cup E\cup S\cup F\\ O_{S} & \text{Set}\;\text{of}\;\text{real}\;\text{container}\;\text{tasks}\;\text{and}\;\text{the}\;\text{dummy}\;\text{starting}\;\text{task},\;O_{S}=I\cup E\cup S\\ O_{F} & \text{Set}\;\text{of}\;\text{real}\;\text{container}\;\text{tasks}\;\text{and}\;\text{the}\;\text{dummy}\;\text{ending}\;\text{task},\;O_{F}=I\cup E\cup F \end{array}$$

(2) Parameters

$$\begin{array}{ll} TT(i,j) & \text{Travel}\;\text{time}\;\text{from}\;\text{physical}\;\text{node}\;\;i\;\text{to}\;\text{physical}\;\text{node}\;j\\ Cap(f) & \text{Maximum}\;\text{number}\;\text{of}\;\text{containers}\;\text{that}\;\text{facility}\;f\;\text{can}\;\text{hold}\;\text{simultaneously}\\ Cap_{f}^{m} & \text{Maximum}\;\text{number}\;\text{of}\;\text{containers}\;\text{that}\;\text{can}\;\text{be}\;\text{accommodated}\;\text{simultaneously}\\ & \text{by}\;\text{each}\;\text{equipment}\;\text{in}\;\text{the}\;m\text{-th}\;\text{equipment}\;\text{active}\;\text{layer}\\ Card(FAC^{m}) & \text{Number}\;\text{of}\;\text{all}\;\text{available}\;\text{equipment}\;\text{in}\;\text{the}\;m\text{-th}\;\text{equipment}\;\text{active}\;\text{layer}\\ a,a_{\left(i,j,t,t^{\prime }\right)} & \text{Space-time}\;\text{active}\;\text{arc}\;\text{that}\;\text{starts}\;\text{at}\;\text{space-time}\;\text{node}\;(i,t)\;\text{and}\;\text{ends}\;\text{at}\;\text{space-time}\;\text{node}\;(j,t\prime )\\ \mu_{n}^{f}(i) & \text{Maximum}\;\text{number}\;\text{of}\;\text{equipment}\;\text{that}\;\text{physical}\;\text{node}\;i\;\text{can}\;\text{hold}\;\text{simultaneously}\\ \mu_{n}^{a_{o}}(i) & \text{Maximum}\;\text{number}\;\text{of}\;\text{transferring}\;\text{active}\;\text{arcs}\;\text{that}\;\text{physical}\;\text{node}\;i\;\text{allows}\;\text{to}\;\text{enter}\;\text{and}\;\text{leave}\;\text{at}\;\text{the}\;\text{same}\;\text{time}\\ \mu_{L}^{f}(i,j) & \text{Maximum}\;\text{number}\;\text{of}\;\text{equipment}\;\text{that}\;\text{physical}\;\text{link}\;(i,j)\;\text{can}\;\text{hold}\;\text{simultaneously} \end{array}$$

$$\begin{array}{ll} HTO(i,k) & \text{Duration}\;\text{of}\;\text{the}\;\text{loading}\;\text{operation}\;\text{when}\;\text{physical}\;\text{node}\;i\;\text{is}\;\text{the}\\ & \text{task}\;\text{start}\;\text{point}\;\text{of}\;\text{the}\;\text{container}\;\text{task}\;\text{k}\;\text{at}\;\text{one}\;\text{equipment}\;\text{active}\;\text{layer}\\ HTD(i^{\prime },k) & \text{Duration}\;\text{of}\;\text{the}\;\text{unloading}\;\text{operation}\;\text{when}\;\text{physical}\;\text{node}\;i\;\text{is}\;\text{the}\\ & \text{task}\;\text{start}\;\text{point}\;\text{of}\;\text{the}\;\text{container}\;\text{task}\;\text{k}\;\text{at}\;\text{one}\;\text{equipment}\;\text{active}\;\text{layer}\\ LT(k) & \text{Duration}\;\text{of}\;\text{the}\;\text{container-lockpin}\;\text{removing}\;\text{or}\;\text{installing}\;\text{operation}\;\text{for}\;\text{container}\;k\;\text{at}\;\text{the}\;\text{transfer}\;\text{platform}\\ O(k)、D(k) & \text{Task}\;\text{start}\;\text{point}\;\text{and}\;\text{task}\;\text{end}\;\text{point}\;\text{of}\;\text{container}\;\text{task}\;k\;\text{in}\;\text{the}\;\text{whole}\;\text{process},\\ & \text{corresponding}\;\text{to}\;\text{the}\;\text{initial}\;\text{container}\;\text{location}\;\text{and}\;\text{the}\;\text{final}\;\text{container}\;\text{location}\\ O^{m}(k)、D^{m}(k) & \text{Task}\;\text{start}\;\text{point}\;\text{and}\;\text{task}\;\text{end}\;\text{point}\;\text{of}\;\text{container}\;\text{task}\;k\;\text{in}\;\text{the}\;m\text{-th}\;\text{equipment}\;\text{active}\;\text{layer}\\ FTS^{M}(k) & \text{Completion}\;\text{time}\;\text{of}\;\text{the}\;\text{unloading}\;\text{operation}\;\text{of}\;\text{export}\;\text{container}\\ & k\;\text{by}\;\text{the}\;\text{main}\;\text{trolley}\;\text{at}\;\text{the}\;\text{designated}\;\text{container}\;\text{location}\;\text{on}\;\text{the}\;\text{ship}\\ FTM^{M}(k) & \text{Completion}\;\text{time}\;\text{of}\;\text{the}\;\text{transport}\;\text{operation}\;\text{of}\;\text{import}\;\text{container}\\ & k\;\text{from}\;\text{the}\;\text{container}\;\text{location}\;\text{on}\;\text{the}\;\text{ship}\;\text{to}\;\text{the}\;\text{transfer}\;\text{platform}\;\text{by}\;\text{main}\;\text{trolley}\\ k,0 & \text{Mark}\;\text{of}\;\text{no-load}\;\text{transport}\;\text{process}\;\text{for}\;\text{container}\;\text{task}\;k,\;\text{when}\;\text{the}\;\text{assigned}\;\text{AGV}\;\text{travels}\;\text{in}\;\text{the}\\ & \text{no-load}\;\text{state}\;\text{from}\;\text{the}\;\text{initial}\;\text{position}\;\text{of}\;\text{receiving}\;\text{the}\;\text{task}\;\text{to}\;\text{the}\;\text{position}\;\text{of}\;\text{loading}\;\text{the}\;\text{container}\\ k,1 & \text{Mark}\;\text{of}\;\text{heavy}\;\text{load}\;\text{transport}\;\text{process}\;\text{for}\;\text{container}\;\text{task}\;k,\;\text{when}\;\text{the}\;\text{assigned}\;\text{AGV}\;\text{travels}\;\text{in}\;\text{the}\\ & \text{heavy}\;\text{load}\;\text{state}\;\text{from}\;\text{the}\;\text{position}\;\text{of}\;\text{loading}\;\text{the}\;\text{container}\;\text{to}\;\text{the}\;\text{position}\;\text{of}\;\text{unloading}\;\text{the}\;\text{container} \end{array}$$

(3) Flow binary decision variables

$$\begin{array}{ll} x_{i,j,t,t^{\prime }}^{k,1} & 0-1\;\text{flow}\;\text{binary}\;\text{decision}\;\text{variable}.\;1,\;\text{if}\;\text{the}\;\text{AGV}\;\text{assigned}\;\text{to}\;\text{container}\;\text{task}\;k\;\text{traverses}\;\text{space-time}\;\text{active}\;\text{arc}\\ & a_{\left(i,j,t,t^{\prime }\right)}\;\text{during}\;\text{the}\;\text{heavy}\;\text{load}\;\text{transport}\;\text{process}\;\text{for}\;\text{container}\;\text{task}\;k\;\text{and}\;0,\;\text{otherwise}.\\ x_{i,j,t,t^{\prime }}^{k,0} & 0-1\;\text{flow}\;\text{binary}\;\text{decision}\;\text{variable}.\;1,\;\text{if}\;\text{the}\;\text{AGV}\;\text{assigned}\;\text{to}\;\text{container}\;\text{task}\;k\;\text{traverses}\;\text{space-time}\;\text{active}\;\text{arc}\\ & a_{\left(i,j,t,t^{\prime }\right)}\;\text{during}\;\text{the}\;\text{no-load}\;\text{transport}\;\text{process}\;\text{for}\;\text{container}\;\text{task}\;k\;\text{and}\;0,\;\text{otherwise}.\\ y_{i,j,t,t^{\prime }}^{f} & 0-1\;\text{flow}\;\text{binary}\;\text{decision}\;\text{variable}.\;1,\;\text{if}\;\text{equipment}\;f\;\text{traverses}\;\text{space-time}\;\text{active}\;\text{arc}\;a_{\left(i,j,t,t^{\prime }\right)}\;\text{and}\;0,\;\text{otherwise}. \end{array}$$

### Model

The objective of the mathematical model is to minimize the operating time of the ship at the dock. The starting time of the operation time is when the ship enters the berth and the container-handling equipment begins operation, while the end time is when the double-trolley quayside cranes finish transferring all the export containers to the designated container location through the unloading operation and finish moving all the import containers from the container location on the ship to the interaction node of the main trolley corresponding to the actual location of the transfer platform.

$$min\;t=max\{max_{k\in E}\{FTS^{M}(k)\},max_{k^{‘}\in I}\{FTM^{M}(k^{‘})\}\}$$ (1)

Constraints (2)-(10) denote the capacity constraints and flow conservation constraints for the non-transferring active arcs. Constraint (8) indicates that for four types of mobile equipment, if one non-transferring active arc is determined to exist, the number of all container tasks corresponding to its space-time link cannot exceed the product of the maximum quantity of the equipment that can be accepted by this space-time link at the same time and the maximum quantity of containers that can be accepted by this equipment at the same time. Constraint (9), similar to constraint (8), states that from the perspective of a non-transferring active arc corresponding to a container task, the number of non-transferring active arcs located on the same space-time link cannot exceed the maximum quantity of equipment that can be accommodated simultaneously by the space-time link. Constraint (10) means that for two types of fixed equipment (including transfer platforms and AGV-mates) that the number of non-transferring active arcs of the container tasks existing at one time in the equipment active layer for each single piece of equipment cannot exceed the maximum quantity of containers that can be simultaneously accommodated by the equipment.

$$\underset{f\in FAC^{m}}{\sum }\underset{\left(i,t)\in In(j,t\prime \right)}{\sum }y_{i,j,t,t\prime }^{f}\leq \mu_{n}^{f}(j),\;\forall j\in N_{\sigma }^{m};\forall m\in \{1,3,4,6\};\forall t\prime \in T$$ (2)

$$\underset{f\in FAC^{m}}{\sum }\underset{\left(i,t)\in In(j,t\prime \right)}{\sum }y_{i,j,t,t\prime }^{f}-\underset{f\in FAC^{m}}{\sum }\underset{\left(i,t)\in Out(j,t\prime \right)}{\sum }y_{i,j,t,t\prime }^{f}=0,\;\forall j\in N_{\sigma }^{m};\forall m\in \{1,3,4,6\};\forall t\prime \in T$$ (3)

$$\underset{f\in FAC^{m}}{\sum }\underset{i\in N_{\sigma }^{m},(i,t)\in Out(D(S),0)}{\sum }y_{D(S),i,0,t}^{f}\leq Card(FAC^{m}),\;\forall m\in \{1,3,4,6\}$$ (4)

$$\underset{k\in Z}{\sum }\underset{\alpha \in \{\text{0},\text{1})}{\sum }\underset{\left(i,t)\in Out(D(S),0\right)}{\sum }x_{D(S),i,0,t}^{k,\alpha }\leq Cap_{f}^{m},\;\forall m\in \{\text{2},\text{5}\};\forall i\in N_{\sigma }^{m}$$ (5)

$$\underset{f\in FAC^{m}}{\sum }\underset{i\in N_{\sigma }^{m},(i,t)\in In(O(F),t\prime )}{\sum }\underset{t\prime \in T\backslash \{1\}}{\sum }y_{i,O(F),t,t\prime }^{f}\leq Card(FAC^{m}),\;\forall m\in \{1,3,4,6\}$$ (6)

$$\underset{\left(i,t)\in In(O(F),t\prime \right)}{\sum }\underset{t\prime \in T\backslash \{1\}}{\sum }x_{i,O(F),t,t\prime }^{F,0}\leq Cap_{f}^{m},\;\forall m\in \{\text{2},\text{5}\};\forall i\in N_{\sigma }^{m}$$ (7)

$$\begin{array}{l} y_{i,j,t,t\prime }^{f}=1\to \sum_{k\in Z}\sum_{\alpha \in \{0,1\}}x_{i,j,t,t\prime }^{k,\alpha }\{\begin{array}{ll} \leq \mu_{L}^{f}(i,j)\cdot Cap(f) & \left(i,j)\in \{(i,j)|i,j\in N_{VI}\right\}\\ =1 & o.w. \end{array},\\ \forall f\in FAC\backslash (FAC^{2}\cup FAC^{5});\forall i,j\in N_{\sigma }(f);\forall t,t\prime \in T,t\neq t\prime ; \end{array}$$ (8)

$$\begin{array}{l} x_{i,j,t,t\prime }^{k,\alpha }=1\to \sum_{f\in FAC(i)}y_{i,j,t,t\prime }^{f}\{\begin{array}{ll} \leq \mu_{L}^{f}(i,j) & \left(i,j)\in \{(i,j)|i,j\in N_{VI}\right\}\\ =1 & o.w. \end{array},\\ \forall k\in O;\forall \alpha =0,1;\forall i,j\in N_{\sigma }\backslash (N_{\sigma }^{2}\cup N_{\sigma }^{5});\forall t,t\prime \in T,t<t\prime ; \end{array}$$ (9)

$$\underset{k\in O}{\sum }\underset{\alpha \in \{\text{0},\text{1}\}}{\sum }\underset{a(i,j,t,t\prime )\in A_{\theta }^{i,t}}{\sum }x_{i,i,t,t\prime }^{k,\alpha }\leq Cap_{f}^{m},\;\forall m\in \{\text{2},\text{5}\};\forall i\in N_{\sigma }^{m};\forall t\in T;$$ (10)

Constraints (11)-(18) describe the flow conservation constraints of the space-time active arcs corresponding to the activities of container transportation, loading and unloading, or lockpin handling, from the perspective of the container task. Constraints (11)-(12) indicate that for each container task, when it is in the equipment active layer related to the main trolley, the portal trolley, the AGV or the ARMG, one equipment is chosen among equipment of the same kind, to transport the container from the task start point to the task end point. Those Constraints also state that the space-time arc related to the unloading activity of the container must appear after that related to the loading activity. Constraints (13)-(14) describe how any piece of mobile equipment or fixed equipment takes on the next container task after completing a task and require that this process is limited by the flow balance between the space-time arcs entering the space-time node and those leaving it. Constraints (15)-(18) indicate that for any space-time node corresponding to the start point, the end point, or the intermediate point of container tasks, the relationship between the numbers of space-time arcs entering the space-time node and those leaving it conform to the logical constraints of the loading and unloading activities.

$$\begin{array}{l} x_{O^{m}(k),O^{m}(k),t,t+HTO(O^{m}(k),k)}^{k,0}\cdot y_{O^{m}(k),O^{m}(k),t,t+HTO(O^{m}(k),k)}^{f}=1\to \\ \sum_{t\prime \in [t+HTO(O^{m}(k),k),t_{\text{max}}]}x_{D^{m}(k),D^{m}(k),t\prime ,t\prime +HTD(D^{m}(k),k)}^{k,1}\cdot y_{D^{m}(k),D^{m}(k),t\prime ,t\prime +HTD(D^{m}(k),k)}^{f}=1,\\ \forall k\in O;\forall f\in FAC^{m};\forall m\in \{1,3,4,6\};\forall t\in T; \end{array}$$ (11)

$$\begin{array}{l} x_{O^{m}(k),O^{m}(k),t,t+HTO(O^{m}(k),k)}^{k,0}=1\to \sum_{t\prime \in [t+HTO(O^{m}(k),k),t_{\text{max}}]}x_{D^{m}(k),D^{m}(k),t\prime ,t\prime +HTD(D^{m}(k),k)}^{k,1}=1,\\ \forall k\in O;\forall m\in \{2,5\};\forall t\in T; \end{array}$$ (12)

$$\begin{array}{l} x_{D^{m}(k),D^{m}(k),t-HTD(D^{m}(k),k),t}^{k,1}\cdot y_{D^{m}(k),D^{m}(k),t-HTD(D^{m}(k),k),t}^{f}=1\to \\ \sum_{k\prime \in O_{F}\backslash \{k\}}\sum_{\left(j,t\prime )\in Out(D^{m}(k),t\right)}x_{D^{m}(k),j,t,t\prime }^{k\prime ,0}\cdot y_{D^{m}(k),j,t,t\prime }^{f}=1,\\ \forall k\in O_{\text{S}};\forall f\in FAC^{m};\forall m\in \{1,3,4,6\};\forall t\in T; \end{array}$$ (13)

$$\begin{array}{l} x_{D^{m}(k),D^{m}(k),t-HTD(D^{m}(k),k),t}^{k,\text{1}}=1\to \sum_{k\prime \in O_{F}\backslash \{k\}}\sum_{\left(j,t\prime )\in Out(D^{m}(k),t\right)}x_{D^{m}(k),j,t,t\prime }^{k\prime ,0}=1,\\ \forall k\in O_{F};\forall m\in \{2,5\};\forall t\in T; \end{array}$$ (14)

$$\begin{array}{l} \sum_{\left(j,t\prime )\in Out(i,t\right)}x_{i,j,t,t\prime }^{k,0}-\sum_{\left(j,t\prime )\in In(i,t\right)}x_{j,i,t\prime ,t}^{k,0}\{\begin{array}{ll} =-1 & i=O^{m}(k,t\in \{t|x_{O^{m}(k),O^{m}(k),t-HTO(O^{m}(k),k),t}^{k,0}=1\}\\ =0 & i=D^{m}(k),t\in \{t|x_{D^{m}(k),D^{m}(k),t-HTD(D^{m}(k),k),t}^{k,1}=1\}\\ =0 & o.w. \end{array},\\ \forall k\in Z;\forall i\in N_{\sigma }^{m};\forall m\in \{1,3,4,6\};\forall t\in T; \end{array}$$ (15)

$$\begin{array}{l} \sum_{\left(j,t\prime )\in Out(i,t\right)}x_{i,j,t,t\prime }^{k,\text{1}}-\sum_{\left(j,t\prime )\in In(i,t\right)}x_{j,i,t\prime ,t}^{k,\text{1}}\{\begin{array}{ll} =1 & i=O^{m}(k),t\in \{t|x_{O^{m}(k),O^{m}(k),t-HTO(O^{m}(k),k),t}^{k,0}=1\}\\ =-1 & i=D^{m}(k),t\in \{t|x_{D^{m}(k),D^{m}(k),t-HTD(D^{m}(k),k),t}^{k,1}=1\}\\ =0 & o.w. \end{array},\\ \forall k\in Z;\forall i\in N_{\sigma }^{m};\forall m\in \{1,3,4,6\};\forall t\in T; \end{array}$$ (16)

$$\begin{array}{l} \sum_{\left(j,t\prime )\in Out(i,t\right)}x_{i,j,t,t\prime }^{k,0}-\sum_{\left(j,t\prime )\in In(i,t\right)}x_{j,i,t\prime ,t}^{k,0}\{\begin{array}{ll} =-1 & i=O^{m}(k),t\in \{t|x_{j\prime ,O^{m}(k),t,t}^{k,1}=1,j\prime \in \{D^{m-1}(k),D^{m+1}(k)\}\}\\ =0 & i=D^{m}(k),t\in \{t|x_{D^{m}(k),j\prime ,t,t}^{k,1}=1,j\prime \in \{O^{m-1}(k),O^{m+1}(k)\}\}\\ =0 & o.w. \end{array},\\ \forall k\in Z;\forall i\in N_{\sigma }^{m};\forall m\in \{\text{2},\text{5}\};\forall t\in T; \end{array}$$ (17)

$$\begin{array}{l} \sum_{\left(j,t\prime )\in Out(i,t\right)}x_{i,j,t,t\prime }^{k,\text{1}}-\sum_{\left(j,t\prime )\in In(i,t\right)}x_{j,i,t\prime ,t}^{k,\text{1}}\{\begin{array}{ll} =1 & i=O^{m}(k),t\in \{t|x_{j\prime ,O^{m}(k),t,t}^{k,1}=1,j\prime \in \{D^{m-1}(k),D^{m+1}(k)\}\}\\ =-1 & i=D^{m}(k),t\in \{t|x_{D^{m}(k),j\prime ,t,t}^{k,1}=1,j\prime \in \{O^{m-1}(k),O^{m+1}(k)\}\}\\ =0 & o.w. \end{array},\\ \forall k\in Z;\forall i\in N_{\sigma }^{m};\forall m\in \{\text{2},\text{5}\};\forall t\in T; \end{array}$$ (18)

Constraints (19)-(26) require non-transferring active arcs and transferring active arcs to meet the capacity constraints and business logic in the container-handling activity. Constraints (19)-(22) require the number of transferring active arcs that link one space-time node of one interaction node or transferring node to meet the capacity limit of the equipment. Constraints (23)-(25) limit the synergy relationship between the transferring active arc and two associated container-handling activity arcs. Constraint (26) limits the sequence logic of three types of activities (including unloading operations, container-lockpin handling operations and loading operations) for any container task on the transfer platform.

$$\underset{k\in Z}{\sum }(\underset{j\in \{O^{m-1}(k),O^{m+1}(k)\}}{\sum }x_{i,j,t,t}^{k,1}+\underset{j\prime \in \{D^{m-1}(k),D^{m+1}(k)\}}{\sum }x_{j\prime ,i,t,t}^{k,1})\leq \mu_{n}^{a_{0}}(i),\;\forall i\in N_{\pi }^{m};\forall m\in \{\text{3},\text{4}\};\forall t\in T;$$ (19)

$$\underset{k\in Z}{\sum }(x_{i,O^{2}(k),t,t}^{k,1}+x_{D^{2}(k),i,t,t}^{k,1})\leq \mu_{n}^{a_{0}}(i),\;\forall i\in N_{\pi }^{\text{1}};\forall t\in T;$$ (20)

$$\underset{k\in Z}{\sum }(x_{i,O^{\text{5}}(k),t,t}^{k,1}+x_{D^{\text{5}}(k),i,t,t}^{k,1})\leq \mu_{n}^{a_{0}}(i),\forall i\in N_{\pi }^{\text{6}};\forall t\in T;$$ (21)

$$\underset{k\in Z}{\sum }(\underset{j\in \{O^{m-1}(k),O^{m+1}(k)\}}{\sum }x_{i,j,t,t}^{k,1}+\underset{j\prime \in \{D^{m-1}(k),D^{m+1}(k)\}}{\sum }x_{j\prime ,i,t,t}^{k,1})\leq \mu_{n}^{a_{0}}(i),\;\forall i\in N_{\sigma }^{m};\forall m\in \{\text{2},\text{5}\};\forall t\in T;$$ (22)

$$x_{D^{m}(k),D^{m}(k),t-HTD(D^{m}(k),k),t}^{k,\text{1}}=1\to x_{D^{m}(k),O^{m}(k),t,t}^{k,\text{1}}=1,\;\forall k\in I;\forall m\in \{1,2,3,4,5\};\forall t\in T;$$ (23)

$$x_{D^{m}(k),D^{m}(k),t-HTD(D^{m}(k),k),t}^{k,\text{1}}=1\to x_{D^{m}(k),O^{m}(k),t,t}^{k,\text{1}}=1,\;\forall k\in E;\forall m\in \{2,3,4,5,6\};\forall t\in T;$$ (24)

$$\begin{array}{l} x_{D^{m}(k),O^{m\prime }(k),t,t}^{k,\text{1}}=1\to x_{D^{m}(k),D^{m}(k),t-HTD(D^{m}(k),k),t}^{k,\text{1}}\cdot x_{O^{m\prime }(k),O^{m\prime }(k),t-HTO(O^{m\prime }(k),k),t}^{k,0}=1,\\ \forall k\in Z;\forall m,m\prime \in \{1,2,3,4,5,6\},m\neq m\prime ;\forall t\in T; \end{array}$$ (25)

$$\begin{array}{l} x_{i,j,t,t}^{k,\text{1}}\cdot x_{j,j\prime ,t\prime ,t\prime }^{k,\text{1}}=1\to \sum_{t\prime \prime \in [t,t\prime -LT(k)]}x_{j,j,t\prime \prime ,t\prime \prime +LT(k)}^{k,\text{1}}=1,\\ \forall k\in Z;\forall j\in N_{\sigma }^{\text{2}};\forall (i,j\prime )\in \{\left(D^{1}(k),O^{3}(k)),(D^{3}(k),O^{1}(k)\right)\};\forall t,t\prime \in T,t<t\prime ; \end{array}$$ (26)

Constraints (27)-(33) restrict the movement of the AGVs in the AGV network, requiring an AGV to avoid the path cycle, opposite-direction link conflicts on two-way roads and crossed link conflicts at intersections and at the same time to satisfy the capacity constraints of the physical nodes. Constraint (27) requires that each physical node is passed through at most once during a no-load or overloaded process for each container task. Similarly, constraint (28) requires that each node from one physical node group at one intersection is passed through at most once during a no-load or overloaded process for each container task. Constraints (29)-(30) require that for the no-load or overloaded process of each container task performed by one of these AGVs, two interaction nodes in the road are separately assigned to the task start point and the task end point and during this process, each node of these two can only be passed through once by this AGV. Meanwhile, other interaction nodes besides these two cannot be passed through by this AGV during this process. Constraint (31) requires that for any two-way road, if there is an equipment active arc on the link of the road, there are no other arcs corresponding to the link in the opposite direction within the time range when the previous arc is operating. Constraint (32) requires that for any intersection, if there is an equipment active arc crossing the intersection, there are no other arcs crossing the same intersection and conflicting with it within the time range when the former arc is operating. Constraint (33) indicates that for space-time nodes corresponding to any interaction node on the AGV road network, the number of non-transferring active arcs that are still active at the time of the space-time node cannot exceed the maximum quantity of equipment that the physical node can hold at the same time.

$$\begin{array}{l} \sum_{t\in T}(\sum_{\left(j,t\prime )\in Out(i,t\right)}x_{i,j,t,t\prime }^{k,\alpha }+\sum_{\left(j,t\prime \prime )\in In(i,t\right)}x_{j,i,t\prime \prime ,t}^{k,\alpha })\leq 2,\\ \forall i\in \Omega_{group}^{w};\forall w\in \{1,\ldots ,W_{group}\};\forall k\in Z;\forall \alpha =0,1; \end{array}$$ (27)

$$\begin{array}{l} \sum_{i\in \Omega_{cluster}\cap \Omega_{group}^{w}}\sum_{t\in T}(\sum_{\left(j,t\prime )\in Out(i,t\right)}x_{i,j,t,t\prime }^{k,\alpha }+\sum_{\left(j\prime ,t\prime \prime )\in In(i,t\right)}x_{j\prime ,i,t\prime \prime ,t}^{k,\alpha })\leq 2,\\ \forall w\in \{1,\ldots ,W_{group}\};\forall k\in Z;\forall \alpha =0,1; \end{array}$$ (28)

$$\begin{array}{l} x_{i,i,t,t+HTO(i,k)}^{k,\text{0}}\cdot x_{j,j,t\prime ,t\prime +HTD(j,k)}^{k,\text{1}}=1\to \\ \sum_{\alpha \prime \in \{0,1\}}\sum_{t\prime \prime \in T}\sum_{i\prime \in N_{\pi }^{4}\backslash \{i,j\}}\left(\sum_{\left(j\prime ,t\prime \prime \prime )\in Out(i\prime ,t\prime \prime \right)}x_{i\prime ,j\prime ,t\prime \prime ,t\prime \prime \prime }^{k,\alpha \prime }+\sum_{\left(j\prime ,t\prime \prime \prime \prime )\in In(i\prime ,t\prime \prime \right)}x_{j\prime ,i\prime ,t\prime \prime \prime \prime ,t\prime \prime }^{k,\alpha \prime }\right)\leq 1,\\ \forall i\in N^{VL};\forall j\in N^{VI};\forall k\in I;\forall t\in T; \end{array}$$ (29)

$$\begin{array}{l} x_{i,i,t,t+HTD(i,k)}^{k,1}\cdot x_{j,j,t\prime ,t\prime +HTO(j,k)}^{k,0}=1\to \\ \sum_{\alpha \prime \in \{0,1\}}\sum_{t\prime \prime \in T}\sum_{i\prime \in N_{\pi }^{4}\backslash \{i,j\}}\left(\sum_{\left(j\prime ,t\prime \prime \prime )\in Out(i\prime ,t\prime \prime \right)}x_{i\prime ,j\prime ,t\prime \prime ,t\prime \prime \prime }^{k,\alpha \prime }+\sum_{\left(j\prime ,t\prime \prime \prime \prime )\in In(i\prime ,t\prime \prime \right)}x_{j\prime ,i\prime ,t\prime \prime \prime \prime ,t\prime \prime }^{k,\alpha \prime }\right)\leq 1,\\ \forall i\in N^{VL};\forall j\in N^{VI};\forall k\in E;\forall t\in T; \end{array}$$ (30)

$$\begin{array}{l} y_{i,j,t,t+TT(i,j)}^{f}=1\to \sum_{f\prime \in FAC^{4}\backslash \{f\}}\sum_{t\prime \in [t+1-TT(j,i),t-1+TT(i,j)]}y_{j,i,t\prime ,t\prime +TT(j,i)}^{f\prime }=0,\\ \forall (i,j),(j,i)\in (N^{VL}\times N^{LC})\cup (N^{TC}\times N^{LC});\forall f\in FAC^{4};\forall t\in T; \end{array}$$ (31)

$$\begin{array}{l} y_{i,j,t,t+TT(i,j)}^{f}=1\to \sum_{f\prime \in FAC^{4}\backslash \{f\}}\sum_{\left(i\prime ,j\prime )\in \Psi_{cluster}^{w,exc}(i,j\right)}\sum_{t\prime \in [t+1-TT(i\prime ,j\prime ),t-1+TT(i,j)]}y_{i\prime ,j\prime ,t\prime ,t\prime +TT(i\prime ,j\prime )}^{f\prime }=0,\\ \forall (i,j)\in \Psi_{cluster}^{w};\forall w\in \{1,\ldots ,W_{cluster}\};\forall f\in FAC^{4};\forall t\in T; \end{array}$$ (32)

$$\underset{f\in FAC^{4}}{\sum }\underset{a_{\left(i,j,t,t\prime \right)}\in A_{\theta }^{i,t\prime \prime }}{\sum }y_{i,i,t,t\prime }^{f}\leq \mu_{n}^{f}(i),\;\forall i\in N_{\pi }^{4};\forall t\prime \prime \in T;$$ (33)

Constraint (34) limits the properties of the decision variables in the model.

$$y_{i,j,t,t\prime }^{f},x_{i,j,t,t\prime }^{k,1},x_{i,j,t,t\prime }^{k,0}\in \{0,1\},\;\forall i,j\in N_{\sigma };\forall k\in Z;\forall f\in FAC;\forall t,t^{\prime }\in T;$$ (34)

## Algorithm

The problem studied in this paper is a combinatorial optimization problem, integrating the subproblems of equipment scheduling, handling lane selection decisions, and AGV conflict-free routing planning, posing a major challenge to the algorithm in terms of the search capability and search efficiency. We proposed a mixed-integer programming model which considers the problem as the extension of the traditional Fixed-charge Capacitated Multicommodity Network Flow Problem. Since many new constraints about collision avoidance are added, the problem in the paper is more complex and harder to solve. SteadieSeifi [34] studied the complexity of this kind of problem and stated that even in small instances, the number of decision variables and constraints was huge, and these numbers would grow rapidly as the scale of the instance increases. The research showed that this kind of problem was difficult to obtain the optimality with current Mixed Integer Programming (MIP) solver, and it mattered to propose an algorithm that could get a better solution in a shorter time.

In previous studies [35–37], the genetic algorithm has been proved to have a strong global search ability and ideal convergence speed for the combinatorial optimization problem and can prevent premature convergence and obtain a better solution combining with the tabu search algorithm. For the scheduling field of container-handling equipment systems, the genetic algorithm and its extensions are the most widely used algorithm [6, 9–11, 18, 23, 38, 39].

**Fig 9:**
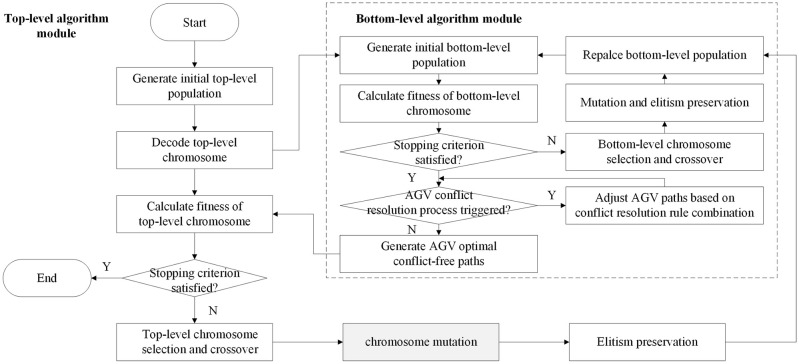
Flow chart of the CRRC-BGA.

**Fig 10:**
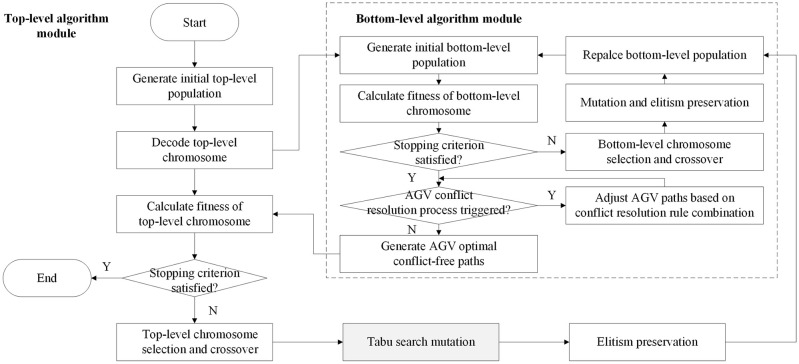
Flow chart of the CRRC-BHGTSA.

In this paper, we propose a bilevel optimization algorithm which integrates genetic algorithm and tabu search algorithm. Since the bilevel genetic algorithm is the most used to solve this kind of problem in previous studies, a bilevel optimization algorithm with a similar algorithm structure, integrating two genetic algorithms, is used as a comparison algorithm to test the performance. Concretely, two bilevel optimization algorithms are designed using the bilevel algorithm framework, namely, the conflict resolution rule combination-based bilevel genetic algorithm (CRRC-BGA) and conflict resolution rule combination-based bilevel hybrid genetic tabu search algorithm (CRRC-BHGTSA). For two bilevel optimization algorithms, the top-level algorithm and the bottom-level algorithm of CRRC-BGA are both genetic algorithms, while those of CRRC-BHGTSA are the hybrid genetic tabu search algorithm and the genetic algorithm, respectively, as shown in Figs 9 and 10. Specifically, the only difference between the two bilevel optimization algorithms is that the former uses conventional genetic mutation operators in the top-level genetic algorithm while in the top-level genetic algorithm of the latter algorithm, the tabu search algorithm is embedded into the mutation link, replacing the normal mutation operation of the genetic algorithm.

Both bilevel optimization algorithms solve integration problems in the top-level algorithmic module, integrate the scheduling of capacitated equipment systems and the selection decision of container-handling lanes, and solve the AGV conflict-free route planning problem on multilane AGV road networks in the bottom algorithm module. The top-level algorithm solves the mathematical model designed in 4.3 and obtains one integrated scheduling scheme. In this scheme, the container scheduling subscheme for AGVs and the handling lane selection subscheme for each container can be transformed into nodes that each AGV passes through and the node sequence corresponding to the travel and can be transmitted to the bottom-level algorithm acting as the decision-making basis. In the bottom-level algorithm, first, the initial shortest path of each AGV is determined by the integrated scheduling scheme of the top-level algorithm; then, AGV conflict judgment is made between any two paths, and conflict resolution rule combination is used to solve different types of AGV conflicts; finally, final conflict-free paths are taken as the AGV optimal path scheme of the optimization result of the top-level algorithm. In each of two bilevel optimization algorithms, the bottom-level algorithm takes the minimization of the total AGV travel time of no-load and overloaded processes for all container tasks as the objective function and needs to satisfy constraints (27)-(30) designed in 4.3, which can be expressed as (35) and (36) here. For any no-load or overloaded process of AGVs, the time to travel from physical node *i* to physical node *j* is represented by *TT*(*i*, *j*). The mathematical model of the bottom-level algorithm is as follows.

$$\text{max}\sum_{i\in N_{\sigma }^{4}}\sum_{j\in N_{\sigma }^{4}}\sum_{t\in T}\sum_{k\in \text{Z}}\sum_{\alpha \in \{\text{0},\text{1}\}}TT(i,j)\cdot x_{i,j,t,t\prime }^{k,\alpha }$$ (35)

s.t.(27)−(30). (36)

### Chromosome representation

**Fig 11:**
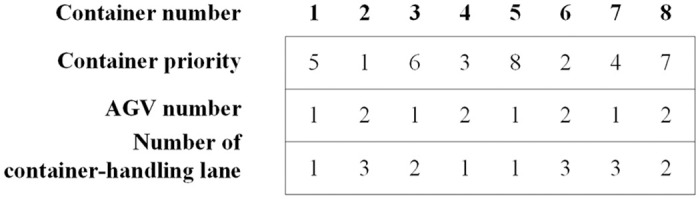
The chromosome representation of the top-level algorithm.

In the chromosome of the top-level algorithm, we use integer coding and design a three-layer chromosome structure. The 1^st^~3^rd^ layers correspond to the operation priority of each container task, the AGV assigned to each container and the container-handling lane assigned to each container, respectively. The container operation in this paper adopts the operating sequence coordination mode of the equipment system to reduce the infeasibility of the scheduling scheme caused by the discordance of the container operation sequence during the equipment operation connection: the main trolley and the portal trolley of the same double-trolley quayside crane have the same working order to the same group of containers; at the same time, the sequence of all DQCs determines the sequence of the AGVs or ARMGs. Fig 11 shows the coding process for a simple problem in which the numbers of DQCs, AGVs, and ARMGs are all 2 and each ARMG serve the import-container storage area or the export-container storage area in the yard. The locations of four import containers on the ship and those of four export containers in the yard, as well as the assigned DQCs and ARMGs, are given. Number 1–4 represent the containers belonging to DQC 1 and number 5–8 represent the containers belonging to DQC 2; number 1, 2, 5 and 6 represent import containers and others represent export containers. The first and second layers of the chromosome indicate the scheduling scheme for all equipment, while the gene value of the third layer of the chromosome represents the serial number of the container-handling lanes corresponding to the eight containers. We take the scheduling scheme of part of the equipment corresponding to the chromosome as an example to illustrate. This chromosome indicates that the operation sequence of DQC 1 is 3-1-4-2, while that of AGV 1 is 5-3-1-7, that of ARMG 1 in the import-container storage area is 5-1-6-2, and that of ARMG 2 in the export-container storage area is 8-3-7-4.

**Fig 12:**
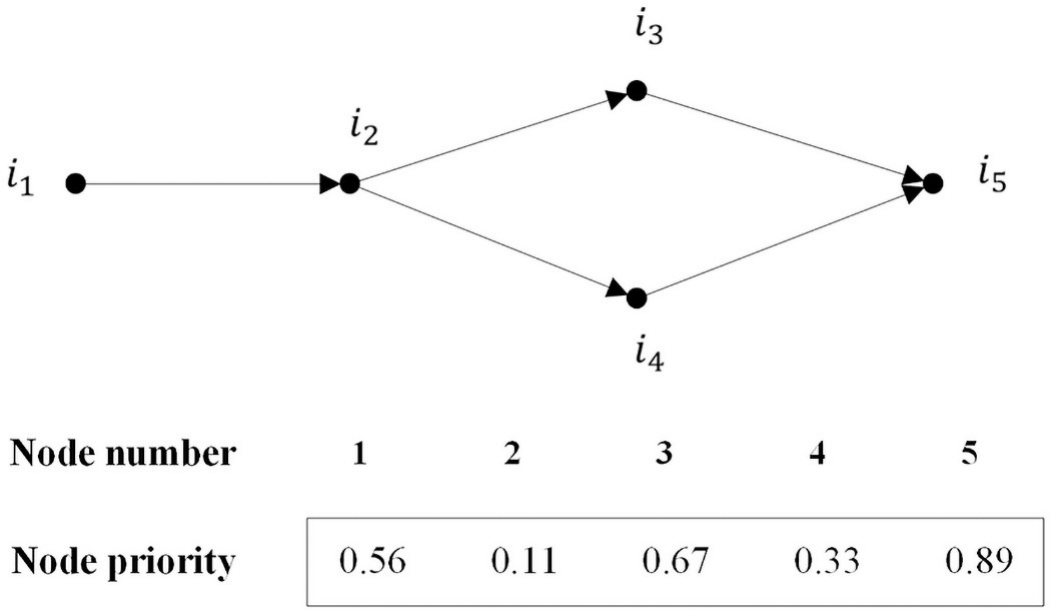
The chromosome representation of the bottom-level algorithm.

In the chromosome of the bottom-level algorithm, we adopt an encoding method based on random number priority with 0–1 non repeated random real numbers. In the chromosome, each element position represents the node number in the road network, and the gene value of each position represents the priority of the node. The node with the highest numerical priority has priority over the others in the same candidate instance. Fig 12 shows the coding process for the nodes in a simple road network with five nodes and one AGV. The start node and the end node of the AGV are node 1 and node 5, respectively. When the AGV reaches node 2, there are two alternatives, namely, node 3 and node 4. Because node 3 has a higher priority, the subsequent path of the AGV is 2-3-5.

### Genetic operators design

(1) Parents selection strategy

‘Roulette wheel’ sampling is used to select individuals from all parental chromosomes at the beginning of each generation in the top-level algorithm or the bottom-level algorithm of both bilevel optimization algorithms. In order to minimize the operating time of the ship at the dock, it is necessary to select the individuals with smaller fitness function.

(2) Genetic crossover operation

In the top-level genetic algorithm, the first layer of the chromosome is crossed by weight mapping crossover and the second and third layers are crossed by two-point crossover. Weight mapping crossover [40] can ensure that offspring inherit the characteristics of their parents, making them suitable for the first layer of chromosome, the gene values of which are not repeated integer priority, with the need to avoid gene value duplications in the cross-generated offspring chromosomes. Two-point crossover [41, 42] randomly selects two tangents in two parental chromosomes and performs content exchange on parental chromosomes. This method is suitable for the second and third layers of the chromosome and can ensure that offspring chromosomes can inherit the feasibility of their parental chromosomes.

In the bottom-level genetic algorithm, the chromosomes are composed of a set of nonrepeating 0–1 random real numbers representing the priority of each node, with two important characteristics, namely, 0–1 random real numbers and nonduplication of gene values, both of which should be taken into account in the process of crossover. We use the classical uniform crossover [42] to cross the chromosome individuals. In the method, the two genes located at the same position on two parental chromosomes are crossed in the order judged by the crossover probability.

(3) Genetic mutation operation

We use the conventional genetic mutation operation in the top-level genetic algorithm and the bottom-level genetic algorithm of the bilevel genetic algorithm and the bottom-level genetic algorithm of the bilevel hybrid genetic tabu search algorithm. After considering the characteristics of the chromosomes of the above algorithms and the application experience in scheduling problems in some related literature, we use swap mutation to mutate the chromosomes of these three genetic algorithms to explore a larger neighborhood space and to prevent the algorithm from falling into one local optimal solution [10, 43].

(4) Tabu search mutation operator

**Fig 13:**
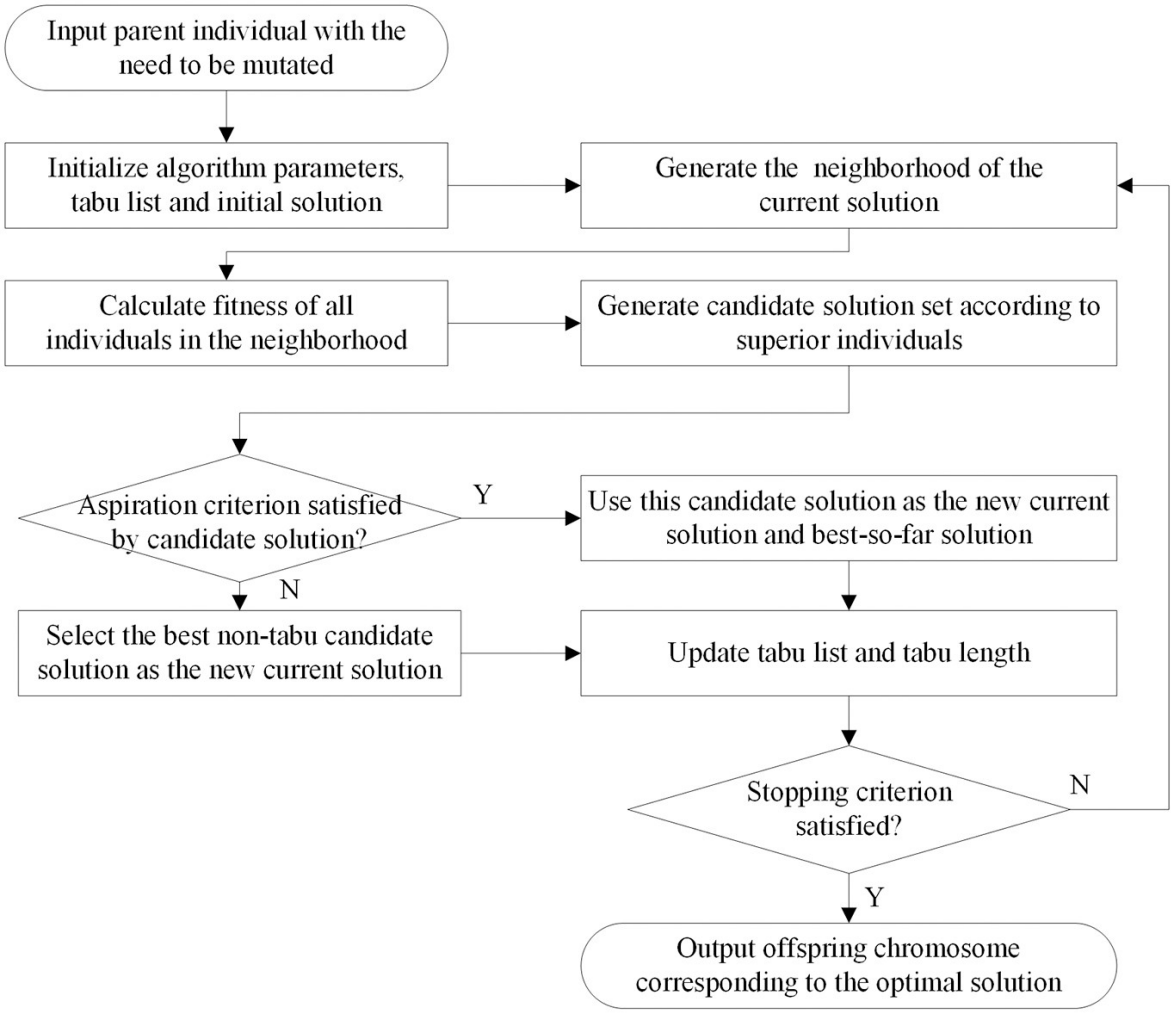
Flow chart of the tabu search mutation operation.

In the top-level genetic algorithm of the bilevel hybrid genetic tabu search algorithm, we use one tabu search mutation operator based on the tabu search algorithm to replace the general genetic mutation operator, which can improve the local search ability of the whole genetic algorithm [37]. The flow of the mutation operation process based on the tabu search mutation operator is shown in Fig 13. First, we judge whether each parent chromosome individual needs to be mutated according to the given mutation probability of the genetic algorithm. Then, the chromosome individuals that need to be mutated are input into the tabu search algorithm and are used as the initial individuals in the process of the tabu search algorithm. Finally, the chromosome corresponding to the optimal solution of the tabu search algorithm is output to the genetic algorithm as the final offspring chromosome of the whole mutation process and continues to be manipulated by subsequent genetic processes.

(5) Elitism preservation strategy

We adopt elitism preservation strategy to screen the parent individuals and the new offspring individuals generated by genetic operations in each generation. The optimal individuals are retained in the new population, according to the fitness function value, to avoid the destruction of the optimal individuals.

### Conflict resolution rule combination based on the multilane AGV road network

**Fig 14:**
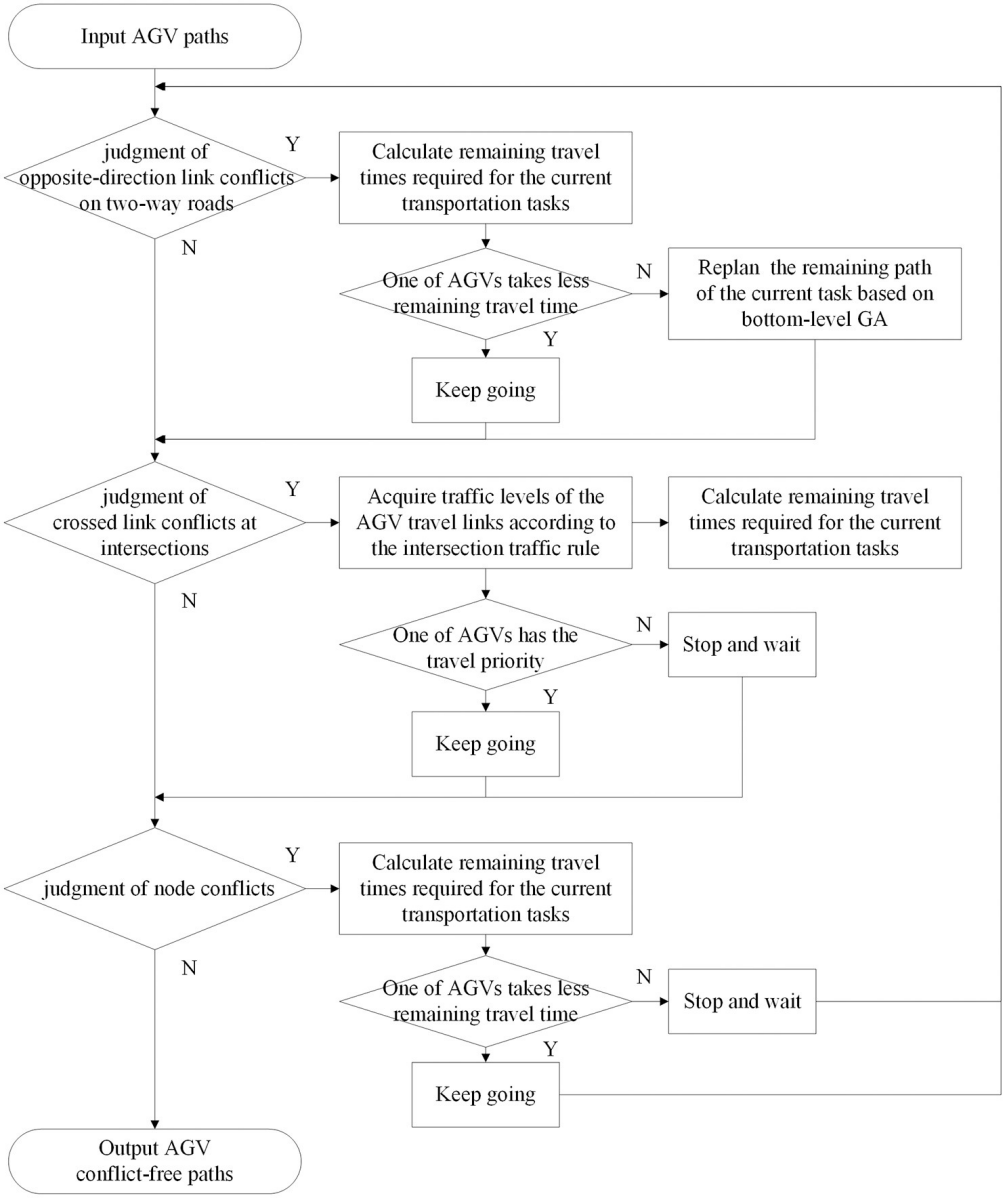
Flow chart of the operation of the conflict resolution rule combination.

In the bottom-level algorithm, a conflict resolution rule combination based on the multilane AGV road network is specially proposed to solve AGV conflicts that occur at actual road network nodes, on two-way roads or at intersections. The rule combination in turn judges the conflict type related to the AGV paths that trigger conflicts according to the order of ’judgment of opposite-direction link conflicts on two-way roads→ judgment of crossed link conflicts at intersections→ judgment of node conflicts ’ and adopts the corresponding conflict resolution rules according to the conflict type, as shown in Fig 14. Among the three types of conflicts, opposite-direction link conflicts on two-way roads often occur in the two-way loading and unloading lane of the container-handling area, which has an important effect on bottlenecks of the quayside cranes in the equipment system. In addition, it is necessary to judge and adjust this type of conflict first because its conflict resolution rule may directly lead to additional AGV conflicts. Similarly, crossed link conflicts at intersections affect the utilization efficiency of intersections and need to be judged and resolved earlier than node conflicts.

**Fig 15:**
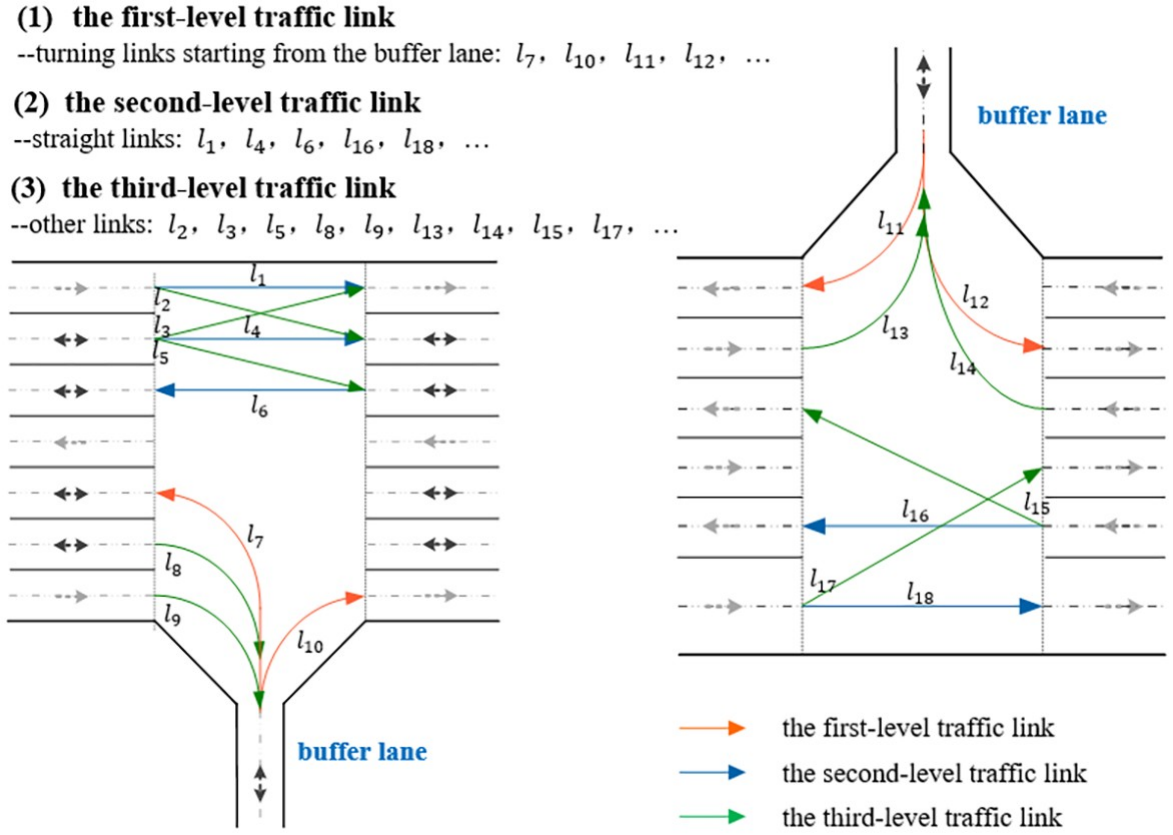
An illustration of the proposed intersection traffic rule.

In the conflict resolution rule combination, a special conflict resolution rule is designed for crossed link conflicts at intersections not studied before in the field of AGV route planning on multilane AGV road networks. The basic idea of this conflict resolution rule is that one AGV continues to travel while another AGV stops and waits, and the core is to determine the AGV with a prior opportunity among all AGVs by judging the priority between the travel levels corresponding to the AGV travel links according to the designed intersection traffic rule [44]. Referring to the intersection traffic rules of urban roads, the new intersection traffic rule classifies traffic links passing through the intersection into three types of traffic links, all of which are set to one certain traffic level, as shown in Fig 15, and requires the AGV that corresponds to the traffic link with the lower level to move forward first. In addition, for two AGVs in crossed link conflict at an intersection and with the same traffic level, this conflict resolution rule requires further comparison between the travel times required for the remaining paths of the current transportation tasks of the two AGVs and provides travel priority to the AGV that takes less time.

### Stopping criterion

Because of the complexity of the model, the algorithm designed in this paper aims to balance the computing time and the difference between the approximate optimal solution and the real optimal solution and uses two stop criterions: (1) the number of iterations of the algorithm reaches the maximum number allowed, and (2) the standard deviation of fitness function values of chromosome individuals in one generation population is less than a small value [45].

## Numerical experiments

In this section, a large number of experiments are carried out to verify the effectiveness of the model, algorithms and AGV conflict resolution rule. In the experiment examples of algorithm verification, 24 basic data combinations involving the number of container tasks and the number of all kinds of equipment are generated, and each combination is input separately into the two bilevel optimization algorithms. Each algorithm is run 20 times with each data combination, and the average objective function values (OFVs) and the average computation time of the algorithm are calculated. In the experimental examples of AGV conflict resolution rule verification, we specifically compare the proposed conflict resolution rule for the crossed link conflicts at intersections with another common conflict resolution rule used for general equipment conflicts in many scenarios. All of the above experiments are performed on a computer with an Intel (R) Xeon (R) CPU E5-2630 v4@2.20 GHz and 504.3 GB of RAM under the Linux operating system.

### Parameter settings

The data used in the experiment mainly correspond to the actual data of the layout and equipment operation of Shanghai Yangshan Port Phase IV automated container terminal, and some of the data are from the research reports and papers of other automated container terminals.

1. The number of container tasks varies from 4 to 40, and that of double-trolley quayside cranes is 2, and that of AGVs varies from 2 to 6, the number of ARMGs varies from 2 to 3, and that of AGV-mates varies from 2 to 3. Each transfer platform of one double-trolley quayside crane is set with two container locations for loading and unloading operations while each AGV-mate is set with five container locations.
2. The scenario of this paper is based on part area of Shanghai Yangshan Port Phase IV automated container terminal, involving the quayside work area, multilane AGV road network and the cantilever-free container storage area of the automated storage yard. In the scenario, there are ten cantilever-free container storage areas and the AGV network is 350m long and 129m wide. To represent this scenario, we design a physical node network mainly including 362 nodes for the multilane AGV road network, 20 nodes for AGV-mate actual positions and 70 nodes for double-trolley quayside cranes.
3. In the actual position of the transfer platform, the time of the integrated handling operation of the main trolley, including one take-off or landing operation and one loading or unloading operation is 25s, the time of the container-lockpin handling operation on the transfer platform is 30s, and the time of the integrated handling operation of the portal trolley, including one take-off or landing operation and one loading or unloading operation is 7s. In the road network, for the AGV, the speed of the travel in a straight line is 6 m/s, while both that in the turning condition and that in the condition of changing lanes in the same direction is 3 m/s. In addition, the turning radius of the travel in the turning condition is not more than 9m. And the time of one loading and unloading operation completed cooperatively by one AGV and one portal trolley at one container-handling lanes is 5s. In the actual position of AGV-mate, the container handling time related to the AGV is 3s, and that by the ARMG is 8s. The travelling time of the main trolley moving between the actual position of the transfer platform and the container location on the ship satisfies the uniform distribution *U*(20,90), and the time of one loading or unloading operation by the main trolley at the container location on the ship is 40s. The travelling time of the ARMG moving between the actual position of the AGV-mate and the container location in the yard satisfies the uniform distribution *U*(30,150), and the time of one loading or unloading operation by the ARMG at the container location in the yard is 5s.
4. Based on the preliminary test, parameters of the algorithm are set as followed: In the genetic algorithm, the crossover rate *P*_*c*_ is 0.85, the mutation rate *P*_*m*_ of 0.08, the population size of 100, and the maximum number of iterations allowed of 80; In the tabu search algorithm, the tabu length is 30 and the maximum number of iterations allowed is 50.

### Results for problems with bilevel optimization algorithms

Table 1 presents the statistical results of the objective function values and the computation times for the bilevel genetic algorithm and the bilevel hybrid genetic tabu search algorithm from 24 small-sized experiments. In the experiments, the number of containers varied from 4 to 40. The statistical data on the computation time show that both bilevel optimization algorithms are completed within an acceptable time and that CRRC-BHGTSA requires more computation time. In addition, the computation time of all the experiments shows an obvious trend. That is, when the number of containers increases, the two algorithms need increasing computation time, and moreover, the computation time required by CRRC-BHGTSA increases much faster.

**Table 1:** Results of computational experiments.

No.	Number of each equipment (DQC-ARMG-AGV)	Containers	CRRC-BGA		CRRC-BHGTSA		OFV gap rate (%)
			OFV (s)	computation time (s)	OFV (s)	computation time (s)	
1	2-2-2	4	470	182	470	267	0.00%
2	2-2-2	6	528	233	528	329	0.00%
3	2-2-3	6	604	317	604	397	0.00%
4	2-2-3	8	689	322	689	447	0.00%
5	2-2-4	8	605	364	605	495	0.00%
6	2-2-4	10	757	418	757	563	0.00%
7	2-2-5	10	739	452	726	589	1.76%
8	2-3-4	10	722	485	696	622	3.60%
9	2-3-3	14	788	502	765	696	2.92%
10	2-3-4	14	754	532	736	730	2.39%
11	2-3-5	14	697	575	686	792	1.58%
12	2-2-4	16	790	596	754	848	4.56%
13	2-3-4	16	810	671	772	884	4.69%
14	2-3-5	16	772	713	744	941	3.63%
15	2-3-6	16	670	784	646	1005	3.58%
16	2-2-4	20	868	878	803	1028	7.49%
17	2-3-5	20	835	938	784	1760	6.11%
18	2-2-3	30	924	1204	839	2801	9.20%
19	2-2-4	30	885	1559	811	4803	8.36%
20	2-2-5	30	871	1980	796	8067	8.61%
21	2-2-4	40	955	2580	924	15101	3.25%
22	2-3-4	40	983	3003	966	28400	1.73%
23	2-3-5	40	946	3991	863	31442	8.77%
24	2-3-6	40	914	5534	825	32616	9.74%

The statistical data of OFV in Table 1 shows that the optimal solution of CRRC-BHGTSA is generally better than that of CRRC-BGA. In the 1^st^~6^th^ experiments, the two bilevel optimization algorithms yield the same optimal solution, and in the 7^th^~24^th^ experiments, the median gap rate between the optimal solutions of the two algorithms is 3.63%.

In conclusion, for the small-sized experiments on the problem, CRRC-BHGTSA can obtain a better approximate optimal solution in the acceptable but more time, and CRRC-BGA has a more obvious advantage in balancing the approximate optimal solution and the computation time.

**Fig 16:**
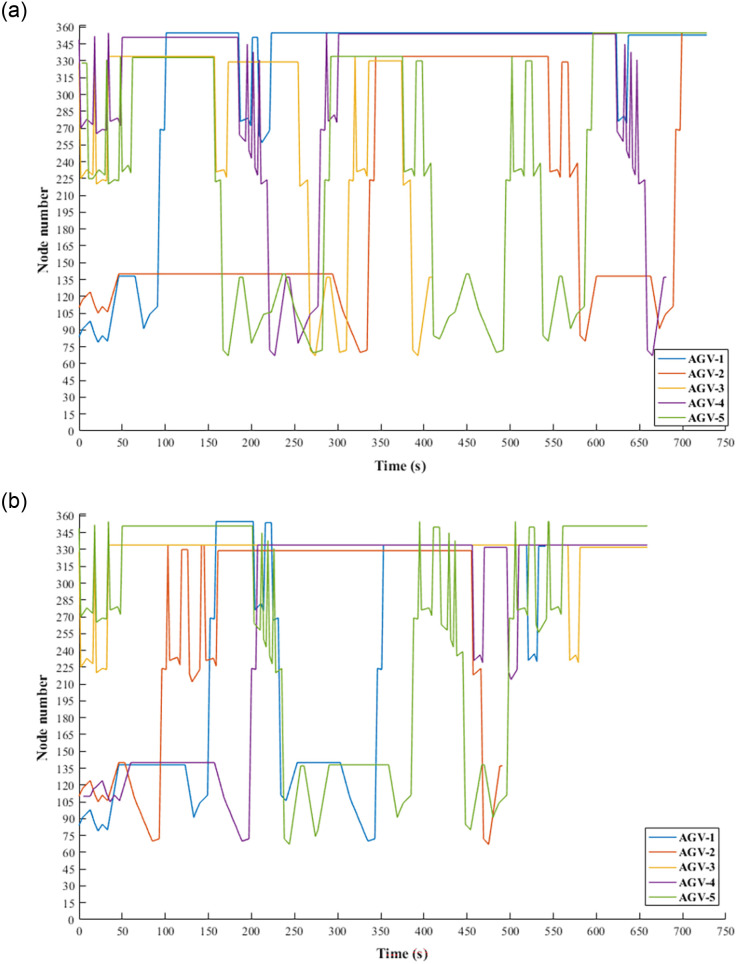
Detailed routing results of the CRRC-BGA and the CRRC-BHGTSA for the 14^th^ experiment. (a) represents the detailed routing result of the CRRC-BGA; (b) represents the detailed routing result of the CRRC-BHGTSA.

Table 1 and Fig 16 show the effect of the numbers of different types of equipment on integrated scheduling. The data in the table show that increasing AGV number has significantly increases the overall operation efficiency of the equipment system and decreases the operation time of the ship on the dock. In each experiment with more than 14 containers, when the number of AGVs is increased, the OFVs obtained by these two bilevel optimization algorithms decrease significantly. In this process, considering the path of AGVs under the 16 container tasks in Fig 16, it can be seen that when the AGV arrives at the loading and unloading node, in most cases, it is necessary for the AGV to wait for the portal trolley that needs to interact with the AGV for the same container task, and there are no multiple AGVs waiting at the same loading and unloading node of the container-handling lane at the same time. This means that this multilane AGV road network has strong abilities to pass and work in cooperation with the double-trolley quayside cranes, which can reduce the node conflict and the opposite-direction link conflict on the two-way road on the two-way container-handling lane when five AGVs simultaneously run on the road network. The multilane network satisfies the work efficiency of the AGV work link and the needs of the whole cooperative work process.

Fig 16 shows the part of the AGV path corresponding to the optimal scheduling scheme of the two bilevel optimization algorithms in the 14th experiment. As seen from Fig 16, the operation link corresponding to the double-trolley quayside crane is the bottleneck of the whole operation process, which causes the AGV that is assigned to operate the same container in the next step to wait in the container-handling lane. This conclusion reflects the actual conditions. However, due to the high equipment acquisition and operating costs of an additional quayside crane, the problem cannot be solved by increasing the number of quayside cranes. In actual operation, only when in the service of medium-sized ships or large ships would the terminal use three or more quayside cranes. In view of the bottlenecks of quayside cranes, terminal operators often adopt the methods of optimizing partial control subsystems of the quayside cranes or upgrading some equipment to improve the operational efficiency of the quayside cranes, such as the addition of a high precision antiswing function and position detection function to the mechanical control system of the main trolley or the replacement of the original manual work with automatic container-lockpin handling equipment on the transfer platform.

**Fig 17:**
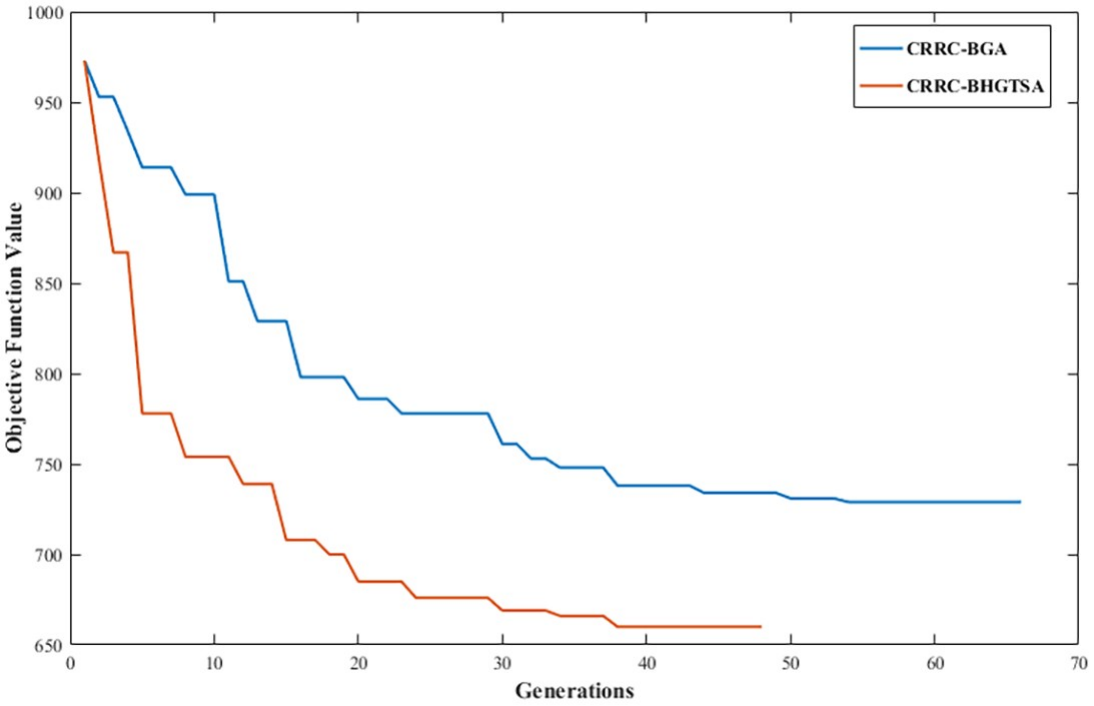
Convergence of the CRRC-BGA and the CRRC-BHGTSA for the 14^th^ experiment.

Fig 17 shows the convergence of the top-level genetic algorithms of the two bilevel optimization algorithms in the 14^th^ experiment. It can be seen from convergence curves that both genetic algorithms achieve convergence within the maximum number of iterations allowed, but the genetic algorithm corresponding to CRRC-BHGTSA shows a faster convergence speed and obtains better results with fewer iterations. This means that the tabu search mutation operator is advantageous in the genetic algorithm compared with the genetic mutation operator and reflects a stronger local search ability.

### Effects of the road intersection conflict resolution rules on the performance

This section introduces verification experiments of the two conflict resolution rules for crossed link conflicts at intersections based on the 14th experiment in section 6.2. In a multilane AGV road network, crossed link conflicts at intersections are a kind of AGV conflict that often occurs during the synchronous operation of multiple AGVs, the solution rule of which is an important factor affecting integrated scheduling. In the conflict resolution rule combination in the multilane AGV road network proposed in this paper, the road intersection traffic rule-based conflict resolution rule (RITR-CRR) is designed to solve the crossed link conflict at intersections. In contrast, the first finish first served-based conflict resolution rule (FFFS-CRR) [46, 47] is a common conflict resolution rule used for conflicts between many types of equipment and does not consider the characteristics of the equipment, travel links or road networks. In the verification experiments, two conflict resolution rules are used separately in the two bilevel optimization algorithms, and then the effect of each algorithm under different intersection conflict resolution rules is compared. For each algorithm with each intersection conflict resolution rule, the problem is solved 30 times, and the median and distribution of the objective function values are calculated. The median indicates the average performance of one algorithm with one certain rule, while the difference between the upper and lower quartiles indicates the fluctuation in the performance.

**Fig 18:**
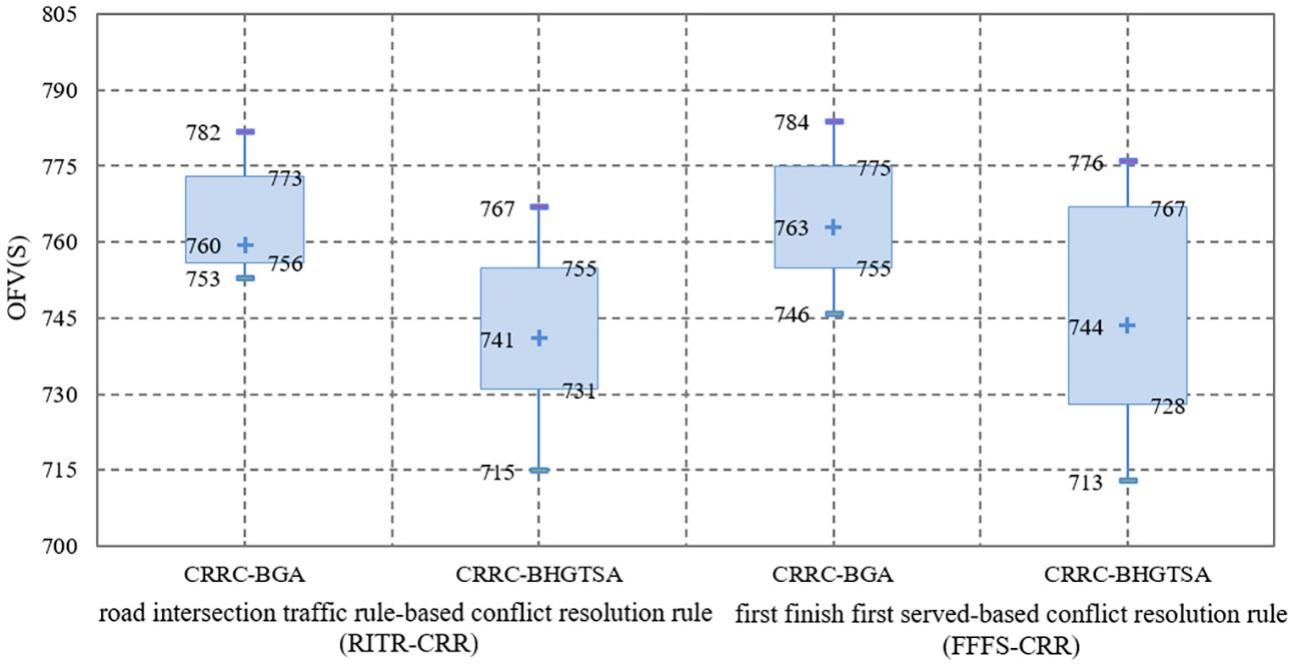
Performance of two bilevel optimization algorithms with each intersection conflict resolution rule for the 14^th^ experiment.

As seen from Fig 18, for the bilevel genetic algorithm CRRC-BGA, the average effect and fluctuation in the algorithms with two different intersection conflict resolution rules are similar, but the algorithm with the RITR-CRR performs slightly better in both aspects. In addition, for the bilevel hybrid genetic tabu search algorithm CRRC-BHGTSA, the average effects of the algorithms with two rules are approximately the same, but the optimal solutions of the algorithm with the RITR-CRR are much less volatile. Generally, considering that the aim of these algorithms is to calculate the approximate optimal solution of the complex model within a finite number of times, the RITR-CRR designed in this paper is more stable and suitable.

## Conclusion

In this paper, the integrated scheduling problem of equipment systems in dual-cycle strategies is studied, which takes into account capacity constraints of the transfer platform and AGV-mate, as well as the AGV conflicts on multi-lane AGV road networks, integrating subproblems of multi-equipment scheduling, handling lane selection decision and AGV conflict-free routing planning. In the problem, a mixed-integer programming model based on the space-time network representation method is designed to describe the complex constraints and the operation logic. Then, to solve the problem, we design two bilevel optimization algorithms based on the conflict resolution rule combination, including the conflict resolution rule combination-based bilevel genetic algorithm (CRRC-BGA) and the conflict resolution rule combination-based bilevel hybrid genetic tabu search algorithm (CRRC-BHGTSA). The experimental results show that both bilevel optimization algorithms can effectively solve the small-sized experiments on the problem where the number of containers varied from 4 to 40 and that each has its own advantages. Generally, CRRC-BGA yields a shorter computation time but has worse searching ability, while CRRC-BHGTSA has stronger searching ability but needs much more computation time. In addition, in the conflict resolution rule combination, the road intersection traffic rule-based conflict resolution rule is proposed to solve the crossed link conflict at intersections and is verified to have strong stability and suitability for the problem through a series of experiments.

Academically, this paper extends the current integrated scheduling problem of terminal equipment system to the integrated scheduling problem of capacitated equipment system with multi-lane road network, and proposes a modeling method based on the space-time network representation method. It is a beneficial attempt. In the practical application, this paper considers many important factors of the actual operation and can be better used to guide the actual work.

In the actual operation of ACTs, integrated scheduling problems with more than one hundred container tasks may need to be solved and require more efficient decision algorithms. The proposed two bilevel optimization algorithms still need to be further improved to obtain better results and reduce the computing time. In the future research, it would be useful to try to design more efficient algorithms by fusing parallel algorithms or other algorithms. In addition, the operation time of container-handling equipment is uncertain under the influence of natural factors or equipment factors and is an important factor affecting the overall operating efficiency. The integrated scheduling problem considering the uncertainty of the job time is an important direction of future research.

## Supporting information

S1 Table Data set of equipment activities. (XLSX)

S2 Table Data set of computational experiments. (XLSX)
